# Regeneration of Capto Core 700 resin through high throughput and laboratory scale studies and impact on production of a SARS‐CoV‐2 vaccine candidate

**DOI:** 10.1002/biot.202200191

**Published:** 2022-07-28

**Authors:** Spyridon Konstantinidis, Seth R. Reinhart, Christine Castagna, Murphy R. Poplyk, Richard R. Rustandi, Kristen L. Flor, Jillian Acevedo‐Skrip, Rachel Thompson, Christopher J. Wang, Sheng‐Ching Wang, Michael A. Winters

**Affiliations:** ^1^ Vaccine Process Research & Development Merck & Co., Inc. Rahway New Jersey USA; ^2^ Vaccine Analytical Research & Development Merck & Co., Inc. Rahway New Jersey USA; ^3^ Investigative Pathology MRL Merck & Co., Inc. Rahway New Jersey USA

**Keywords:** Capto Core, chromatography, CIP, high throughput, live virus vaccine, RoboColumns, scale‐up

## Abstract

During the development of a SARS‐CoV‐2 vaccine candidate, at the height of the COVID‐19 pandemic, raw materials shortages, including chromatography resins, necessitated the determination of a cleaning in place (CIP) strategy for a multimodal core‐shell resin both rapidly and efficiently. Here, the deployment of high throughput (HT) techniques to screen CIP conditions for cleaning Capto Core 700 resin exposed to clarified cell culture harvest (CCCH) of a SARS‐CoV‐2 vaccine candidate produced in Vero adherent cell culture are described. The best performing conditions, comprised of 30% n‐propanol and ≥0.75 N NaOH, were deployed in cycling experiments, completed with miniature chromatography columns, to demonstrate their effectiveness. The success of the CIP strategy was ultimately verified at the laboratory scale. Here, its impact was assessed across the entire purification process which also included an ultrafiltration/diafiltration step. It is shown that the implementation of the CIP strategy enabled the re‐use of the Capto Core 700 resin for up to 10 cycles without any negative impact on the purified product. Hence, the strategic combination of HT and laboratory‐scale experiments can lead rapidly to robust CIP procedures, even for a challenging to clean resin, and thus help to overcome supply shortages.

## Graphical Abstract and Lay Summary

A chimeric SARS‐CoV‐2 live virus vaccine candidate (VSVΔG‐SARS‐CoV‐2) was purified via Capto Core 700 chromatography. High Throughput (HT) scale column chromatography was deployed to screen cleaning in place (CIP) agents and to test a CIP strategy for 10 cycles. This involved the determination of product purities and yields and of the presence of foulants post CIP in resin extracts from miniature (600 μL) columns between cycles. The results were verified at 20 mL column scale for 10 batches. This work demonstrates that HT scale techniques can lead to effective CIP strategies for chromatography resins.

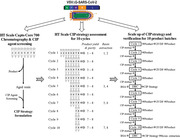

**Abbreviations**
Au: absorbance units
BSA: bovine serum albumin
CCCH: clarified cell culture harvest
ChromP: chromatography flowthrough product pool
CIP: Cceaning in place
CIP1: cleaning in place step 1
CIP2: cleaning in place step 2
CIP3: cleaning in place step 3
CV: column volume
DBC_10%_
: dynamic binding capacity at 10% breakthrough
DF: diafiltration
FBS: fetal bovine serum
FT: flowthrough (effluent collected during column loading)
G: V vesicular stomatitis virus glycoprotein
hcDNA: host cell DNA
HCP: host cell proteins
HT: high throughput
L: vesicular stomatitis virus large polymerase
LMH: liters per square meter hour
LOQ: limit of quantitation
LVV: live virus vaccine
M: vesicular stomatitis virus matrix protein
N: vesicular stomatitis virus nucleoprotein protein
P: vesicular stomatitis virus phosphoprotein
PAGE: polyacrylamide gel electrophoresis
PBS: phosphate buffered saline
PCIP: post cleaning in place
Pen Strep: Penicillin‐Streptomycin
qPCR: quantitative polymerase chain reaction
RC: RoboColumn
S: SARS‐CoV‐2 spike glycoprotein
SDS: sodium dodecyl sulfate
TEM: transmission electron microscopy
UF: ultrafiltration
UFCR: ultrafiltration concentrated retentate
UFDR: ultrafiltration diafiltrated retentate
UFP: ultrafiltration product
*V_10%_*
: 10% breakthrough volume
*V_o_*
: column void volume
VSV: vesicular stomatitis virus
*ρ*
: Spearman's rank correlation coefficient

## 1 INTRODUCTION

Cost of goods for bioprocess is typically dominated by downstream processing^[^ ^1^ ^]^ and hence the implementation of cleaning in place (CIP) techniques for chromatography resins is important for mitigating the costs associated with the use of this unit operation.^[^ ^2^ ^]^ The development of CIP strategies for biopharmaceuticals, such as monoclonal antibodies, has been reported for affinity, ion exchange, and hydrophobic interaction‐ion exchange (e.g.,^[^ ^3^, ^4^, ^5^ ^]^) resin modalities. This typically includes the deployment of multiple CIP agents, such as high conductivity and caustic solutions, aiming to remove tightly bound residuals from the resin that would otherwise lead to its fouling and to a potentially significant reduction of the resin's performance in purifying a target product over multiple cycles. The number of cycles can vary from small to large in batch and continuous processes^[^ ^6^ ^]^ and column re‐use in such processes requires the completion of studies validating the lifespan of chromatography media.^[^ ^7^ ^]^

Apart from cost savings, the re‐use of chromatography resins can be a necessity in situations wherein supply limitations are in place. This was the case during the development of a SARS‐CoV‐2 vaccine candidate at the height of the pandemic. The processing of live virus vaccines (LVVs) often requires the purification of large targets (>100 nm in diameter) that are more complex than many recombinant subunit protein therapeutics counterparts.^[^ ^8^, ^9^, ^10^, ^11^ ^]^ LVV size, along with the accrued avidity of interactions with functionalized stationary phases, often leads to low binding capacities and recoveries when purified via bind and elute chromatography. Hence, chromatography steps run in flowthrough mode may be preferred. Capto Core resin technology (Cytiva, Uppsala, Sweden) provides a unique mode of separation for the purification of LVVs. Here, the inactive outer shell acts as a sieve allowing solutes below a molecular weight cut‐off to diffuse into and bind to the functionalized inner bead, which displays a triple mode of action due to the octylamine ligand. Consequently, LVVs will flow through, and impurities will be removed from the product pool by adsorbing to the resin. This mode of separation has made the Capto Core resins highly desirable for LVV processing, including SARS‐CoV‐2 vaccines.^[^ ^12^ ^]^ This, and its application for the purification of additional vaccine products^[^ ^13^, ^14^ ^]^ led to uncommonly severe supply shortages during the COVID‐19 pandemic.

The CIP of the Capto Core 700 resin is expected to be challenging since the nature of its functionalized inner core can lead to irreversible binding of solutes that may not be easily interrupted.^[^ ^15^ ^]^ This challenge is further compounded in that the resin is often applied in flowthrough mode, typically as the first step in a purification process following primary recovery. Hence, a higher content of diverse solutes bind to the resin and require removal prior to resin re‐use. Here, a methodology for screening cleaning agents and testing cleaning strategies for Capto Core 700 resin, exposed to clarified cell culture harvest (CCCH) expressing a replication‐competent chimeric SARS‐CoV‐2 LVV candidate, is presented. Experiments were completed at microscale and laboratory scales to ultimately determine the feasibility of CIP and re‐using a Capto Core 700 column during the production of batches of the LVV candidate. Miniature column chromatography, with the use of RoboColumns (e.g.,^[^ ^16^ ^]^), was employed to design a multi‐step CIP strategy involving cleaning agents disrupting the binding of solutes to the resin. Leading candidate CIP agents were then deployed in resin re‐use experiments, also performed using RoboColumns. Here, the performance of the CIP strategy was assessed across ten cycles by tracking multiple outputs, such as chromatographic traces, product and impurity flowthrough yields, and by determining directly the presence of bound solutes post‐CIP in resin extracts using a procedure that combined and expanded on earlier approaches.^[^ ^17^, ^18^ ^]^ The results from the microscale experiments were verified at the lab scale where resin re‐use experiments were also performed by scaling up the CIP strategy. While the same rigorous analysis was applied to the scale‐up experiments, for confirming the absence of a negative impact of the CIP strategy on the chromatography step, here a holistic approach was adopted by characterizing the impact of the resin's re‐use on the entire purification process generating the final purified product. It is shown that the microscale experiments are scalable and the formulated CIP strategy can be adopted to re‐use the Capto Core 700 resin for the production of a SARS‐CoV‐2 vaccine candidate without any adverse impact on the delivered purified product. The combination of microscale and lab‐scale experiments can, therefore, determine the feasibility of CIP for a challenging to clean resin, exposed directly to CCCH for the production of an LVV. This leads to the highly desirable mitigation of costs and supply limitations.

## 2 EXPERIMENTAL SECTION

### 2.1 Chimeric VSV∆G‐SARS‐CoV‐2 virus production

Replication‐competent, chimeric VSV∆G‐SARS‐CoV‐2 LVV candidate was generated by replacing the live vesicular stomatitis virus (VSV) glycoprotein (G) gene with a coding sequence for the SARS‐CoV‐2 spike glycoprotein (S).  VSV∆G‐SARS‐CoV‐2 was produced in Vero cells. Cell suspensions were grown in a 250 L single‐use bioreactor (SUB) (Thermo Fisher Scientific Inc., MA, USA) containing Cytodex‐1 gamma‐irradiated microcarriers (Cytiva) and GIBCO VP‐SFM media (Thermo Fisher Scientific Inc.), supplemented with L‐glutamine and P‐188. Prior to infection with VSV∆G‐SARS‐CoV‐2 virus, a media exchange was performed using fresh GIBCO VP‐SFM media, supplemented with L‐glutamine and P‐188. Approximately 48 h post‐infection, the SUB was harvested using a Harvestainer microcarrier separation system (Thermo Fisher Scientific Inc.). This took place after the addition of Benzonase Endonuclease (MilliporeSigma, MA, USA) and magnesium chloride directly to the SUB, to final concentrations of 60 U mL^–1^ and 2 mM, respectively, 90 min prior to the reactor's harvest. The cell culture harvest was then clarified using a Sartoclean CA 3 μm|0.8 μm filter (Sartorius Stedim Biotech GmbH, Goettingen, Germany). CCCH, containing the expressed VSV∆G‐SARS‐CoV‐2 vaccine candidate, was either processed further immediately upon its generation or aliquoted and stored at –70°C until further use.

### 2.2 High throughput chromatography

#### 2.2.1 Robotic station

High throughput (HT) chromatography studies using PreDictor RoboColumns (Cytiva, Uppsala Sweden), packed with either 200 or 600 μL of Capto Core 700 resin (Cytiva), were carried out based on the method described in Ref. [16] Here, a Tecan Freedom EVO 150 robotic station was employed, which was controlled by Freedom EVOware v2.8 (Tecan Group Ltd., Männedorf, Switzerland) run on an Intel i5 4670 CPU machine with 4 GB of RAM running Windows 7 (Microsoft Corporation, WA, USA). The robot was equipped with an 8‐channel liquid handling arm and an eccentric robot manipulator arm. Moreover, it was integrated with an Infinite M1000 Pro reader, operated by i‐control software, (Tecan Group Ltd.), and an Agilent Velocity 11 VSpin with an access centrifuge (Agilent Technologies, CA, USA). The liquid handling arm was connected to 1 mL syringes with a conical cap and short uncoated, stainless steel tips. Standard tubing was used throughout. For the completion of RoboColumn experiments, the robot was also equipped with the Te‐Shuttle and Te‐Chrom modules (Tecan Group Ltd.). The deck layout of the method running the RoboColumn experiments (Figure S1, Supporting Information) remained largely unchanged regardless of the use of different types of collection plates (microplates or deep well plates) and RoboColumn sizes. In particular, the required changes were limited to adjusting the height of the Te‐Chom module, to accommodate deep well collection plates, and the use of re‐purposed 9‐site Hotels (Tecan Group Ltd.) which allowed for the storage of up to four deep well collection plates, instead of conventional nine, with or without lids. Additional details can be found in Figure S1, Supporting Information.

#### 2.2.2 CIP high throughput screening experiments

For the screening of CIP agents, 200 μL RoboColumns packed with Capto Core 700 were employed. The columns were equilibrated with five column volumes (CVs) of a 10 mM Tris, pH 7.5, 150 mM NaCl buffer, and with a residence time of 2 min. Post equilibration, the columns were loaded directly with CCCH for 70 CVs with a residence time of 4 min and washed with the equilibration buffer for 5 CVs and with a residence time of 2 min. The washing of the columns was followed by their CIP with the sequential deployment of up to 3 CIP steps, termed CIP1, CIP2, and CIP3, respectively, where each CIP step utilized a different solution. CIP1–CIP3 were applied to the columns for a duration of 7 CVs with a 4 min residence time unless CIP2 was comprised of a water flush in which case CIP2 was applied for 2 CVs. In these steps, a collection of cleaning agents was tested including acids (acetic acid), bases (NaOH), solvents (ethanol, isopropanol, and n‐propanol), chaotropes (urea), and combinations of thereof. Post application of CIP1–CIP3, the columns were then subjected to a post‐CIP flush with equilibration buffer for 5 CVs and at a residence time of 2 min. All chemicals were of analytical grade and acquired from Sigma‐Aldrich (MO, USA) unless stated otherwise. While loading, washing, and cleaning columns, effluent fractions of 0.6–1.8 mL were collected in Axygen 2.2 mL 96‐well deep square well collection plates (Corning Life Sciences, MA, USA). These were either assayed immediately upon the completion of experiments or sealed with Thermo Scientific Nunc sealing tape (Thermo Fisher Scientific Inc., MA, USA) and stored at 4°C until analytical testing. Alternatively, pools of fractions were aliquoted in 0.5 mL Matrix 2D barcoded tubes (Thermo Fisher Scientific Inc.) and stored at –70°C. Where desired, chromatograms were generated by transferring 150 or 200 μL of the collected fractions to half‐ or full‐area UV transparent microplates, respectively, (Corning Life Sciences) and their absorbance was measured on the integrated Infinite M1000 Pro reader at 280 nm. Measurements at 900 and 990 nm were also made for path length correction purposes.^[^ ^19^ ^]^

#### 2.2.3 Resin CIP and high throughput scale resin re‐use experiments

**FIGURE 1:**
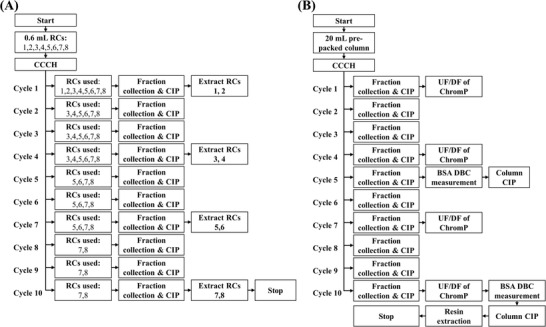
Design of studies for implementing resin re‐use experiments while purifying the VSV∆G‐SARS‐CoV‐2 vaccine candidate from clarified cell culture harvest (CCCH) and cleaning in place (CIP) the used Capto Core 700 columns at (A) High throughput scale using eight RoboColumns (RCs); and (B) Lab‐scale using a 20 mL pre‐packed column. In (B) the chromatography flowthrough product pool (ChromP) was further processed via ultrafiltration/diafiltration (UF/DF) at cycles 1, 4, 7, and 10 and the column was used to measure the dynamic binding capacity (DBC) of bovine serum albumin (BSA) at the end of cycles 5 and 10

The robotic system was also deployed to run multi‐cycle HT scale purifications of the VSV∆G‐SARS‐CoV‐2 vaccine candidate with resin CIP between each cycle. A total of 10 cycles were performed (Figure 1A), since they represented an estimate of the minimum number of batches of the VSV∆G‐SARS‐CoV‐2 vaccine candidate produced annually without parallel processing, with eight 600 μL Capto Core 700 RoboColumns (RC1–RC8). Fractions were collected in Axygen 2.2 mL 96‐well deep square well plates. Here, at the beginning of each cycle, the columns were flushed with system liquid for 5 CVs to remove the storage solution. This was followed by their equilibration with 10 CVs of 10 mM Tris, pH 7.5, and 150 mM NaCl with a residence time of 2 min. The columns were then loaded with 70 CVs of CCCH with a residence time of 6 min and fractions were collected every 1.75 mL. Following this, the columns were washed with the equilibration buffer for 1 CV with a 6 min residence time while collecting the effluent in a 0.6 mL fraction. The columns were then cleaned in place in three sequential steps (i.e., CIP1 – CIP3), each applied with a residence time of 6 min while collecting 0.6 mL fractions. CIP1, CIP2, and CIP3 were applied for 3, 5, and 3 CVs, respectively.

At the end of each of cycles 1, 4, 7, and 10, two columns were removed at a time from further cycles (Figure 1A); one was used for resin extraction, shortly after the completion of the experiment, and the second was sealed and stored at 4°C. Hence, from RoboColumns 1–8, only RCs 7 and 8 were used across all 10 cycles. For cycles 1, 4, 7, and 10, those columns that were to be removed from further cycling experiments (e.g., RCs 1 and 2 at end of cycle 1), were also flushed with 5 CVs of equilibration buffer, following CIP3, with a residence time of 6 min and while collecting 1.5 mL fractions. Alternatively, those RoboColumns that were also tested in subsequent cycles were stored in 1 N NaOH (i.e., CIP3) until their use. At the end of each cycle, the fractions collected from each RoboColumn during their loading and CIP1–CIP3 applications were combined into half‐ or full‐area UV transparent plates, or Matrix 2D barcoded tubes, to create separate pools by mixing equal size aliquots. The pools, fractions, and resin extracts were stored at 4°C prior to their analysis or at –70°C for long‐term storage. Chromatograms were generated by aliquoting up to 200 μL of fraction volumes into half‐ or full‐area UV transparent plates and measuring their absorbance at 280, 900, and 990 nm.

### 2.3 Lab‐scale resin re‐use experiments

Lab‐scale resin re‐use studies (Figure 1B) were performed by employing an OPUS ValiChrom column with 11.3 mm internal diameter and 200 mm bed height (Repligen, MA, USA), prepacked with 20 mL of Capto Core 700. It was connected to an ÄKTA Pure chromatography system, operated by UNICORN v7 (Cytiva). For each cycle, the column was first flushed with 3 CVs of 0.5 N NaOH for a total of 12 min and then equilibrated for 10 CVs with a 10 mM Tris, 150 mM NaCl, pH 7.5 buffer, and with a residence time of 2 min. The column was then loaded with 65 CVs of CCCH (6 min residence time) and washed for 2 CVs with the equilibration buffer (6 min residence time). Following this, the column was cleaned in place by the application of CIP agents in three steps (CIP1, CIP2, and CIP3) with durations of 3, 5, and 3 CVs, respectively, and each with a residence time of 6 min. The effluent streams generated while loading, washing, and CIP of the column were collected in separate Nalgene™ bottles (Thermo Fisher Scientific Inc.). Aliquots of these were transferred to Matrix 2D barcoded tubes and stored at –70°C until analytical testing. In addition to using the column to purify the VSV∆G‐SARS‐CoV‐2 vaccine candidate in ten cycles, the cleaned column was also used to measure the dynamic binding capacity of bovine serum albumin (BSA) at the end of cycles 5 and 10 (Figure 1B).

#### 2.3.1 BSA dynamic binding capacity measurements

To determine the dynamic binding capacity of BSA at 10% breakthrough (*DBC_10%_*) the cleaned in place 20 mL column (Figure 1B) was flushed with 0.5 N NaOH for 3 CVs at a residence time of 4 min and then equilibrated with 10 mM Tris, 150 mM NaCl, pH 7.5 for 10 CVs at a 2 min residence time. Following this, the column was loaded with a 2.74 g L^–1^ BSA solution, prepared in equilibration buffer, with a residence time of 2 min. A breakthrough was monitored by observing the absorbance trace at 280 nm and comparing it against its value at 100% breakthrough. The *DBC_10%_* for BSA was estimated based on Equation (1) where *V_10%_* and *V_0_* are the breakthrough volume at 10% and column void volume, respectively. The same process was followed to measure *DBC_10%_* on separate, fresh 20 mL columns to obtain a reference *DBC_10%_* for BSA on Capto Core 700 at the same mobile phase and residence time conditions.

(1) $$DBC_{10\%}\;=\;2.74\times \left(V_{10\%}-V_{0}\right)/20$$

#### 2.3.2 Ultrafiltration/Diafiltration

Frozen Capto Core 700 flowthrough product pools from cycles 1, 4, 7, and 10 were thawed for use in Ultrafiltration/Diafiltration (UF/DF) experiments using separate polysulfone hollow fiber membrane cartridges with a 500 kDa nominal molecular weight cut‐off pore size, 0.5 mm diameter lumen, and 140 cm^2^ membrane area (Cytiva). The cartridges were operated at 2–8°C under constant crossflow and permeate fluxes of 1080 LMH and 44.3 LMH, respectively. Each flowthrough product pool was loaded at 92.9 L m^–2^, concentrated by reducing the batch volume by a factor of 25 (UFCR), and then diafiltered against 8 diafiltration volumes of a 10 mM Tris, 150 mM NaCl, 10 g L^–1^ rHSA pH 7.5 buffer (UFDR). The final product was then further concentrated by reducing the batch volume by an additional factor of 2 (UFP). Intermediates and final products were sampled, and aliquots were collected in Matrix 2D barcoded tubes which were stored at –70°C until analytical testing.

### 2.4 Resin extraction

Capto Core 700 resin in 200 and 600 μL RoboColumns, which had been previously exposed to CCCH and subsequently cleaned in place, was extracted by removing the resin from the column housing and treating it with a combination of a reducing agent and a detergent. For this purpose, the top cover of the columns was removed, and the housing, containing the resin, was placed upside down inside a pre‐weighted Corning Falcon 15 mL conical centrifuge tube (Corning Life Sciences). For 600 μL columns, the housing was first placed upside down in a weighted 1.5 mL Thermo Scientific Nalgene cryogenic tube (Thermo Fisher Scientific Inc.) before they were both transferred to the 15 mL tube. The 15 mL conical tubes were then centrifuged at 500 g for 5 min using a Sorvall Legend XTR centrifuge (Thermo Fisher Scientific Inc.). The column housing was then removed from the centrifuge tube, and the tube containing the resin was weighed. For 600 μL columns, once the column housing was removed, the cryogenic vial itself, containing the resin, was also removed from the 15 mL centrifuge tube and weighted. Following this, the collected resin was washed once with a freshly prepared 27.8 mM DTT/17.3 mM SDS solution which included 24.8 mM Tris. For 200 μL columns, the solution was added directly to the 15 mL tube to generate a 25% slurry and its addition was followed by a brief mixing and a centrifugation step at 500 g for 5 min to remove the supernatant. For 600 μL columns, the DTT/SDS solution was added directly to the cryogenic tube in three 0.8 mL steps. After each addition, the cryogenic tube was mixed briefly and the slurry was transferred to the 15 mL centrifuge tube. Once the 25% slurry was collected in the centrifuge tube it was mixed briefly and centrifuged at 500 g for 5 min to remove the supernatant. Upon the completion of the wash, the DTT/SDS solution was added one last time to the resin. The resulting 25% slurry was mixed briefly, and an aliquot of 0.5 mL was taken shortly after. For both RoboColumn sizes, the 15 mL centrifuge tube was weighted after each generation of the 25% slurry and removal of the supernatant. The slurry aliquot was transferred to a 1.5 mL Eppendorf Safe‐Lock tube (Eppendorf, NY, USA), which was loaded to a Mastercycler Gradient (Eppendorf, NY, USA) and heated at 104°C for 10 min. The treated resin was mixed briefly and centrifuged at 5000 g for 5 min, and the supernatant, termed henceforth as extract, was collected carefully without disrupting the resin pellet. The extract was kept at 4°C over short periods prior to its analysis. The same procedure was also implemented for extracting the Capto Core 700 resin from the 20 mL lab‐scale pre‐packed column at the end of the last resin re‐use cycle. Here, the top of the column was removed, and the resin was gently pushed out from the bottom of the column. The top, middle, and bottom portions of the resin bed were collected as separate layers, each corresponding to a third of the bed height/volume. The layers were then extracted either separately, or in their combination, by mixing equal size aliquots, to represent the entire bed.

### 2.5 Analytical methods

#### 2.5.1 Quantitative western blotting

CCCH, fraction pools, and resin extracts were tested with a panel of quantitative western blotting analytics run on Wes, Jess, Sally Sue, and/or Peggy Sue systems (Protein Simple, CA, USA) employing the appropriate selection of size‐based assay kits testing for both protein impurities and product. Unless stated otherwise, all reagents, modules, and kits were obtained from Protein Simple and the latter two were run as per the supplier's instructions. For all assays used, pre‐diluted samples were denatured with a master mix solution in a Thermo Scientific Armadillo PCR 96‐well plate (Thermo Fisher Scientific Inc.) for 5 min at 95°C using a Mastercycler Gradient (Eppendorf). Denatured samples were then loaded into an instrument‐specific plate as per the supplier's recommendations. The prepared plates were then transferred to a given instrument for automated capillary electrophoresis analysis. For all analyses, the generated peaks were evaluated in Compass for SW (Protein Simple) using default baseline settings and a dropped lines peak integration with threshold and width values of 10.0 and 12.0, respectively. Details on the individual product and impurity assays, including exposure settings, are as follows.

##### 2.5.1.1 Product assays

VSV∆G‐SARS‐CoV‐2 LVV product was tracked by analyzing samples for their content in nucleoprotein (N), specific for VSV,^[^ ^20^ ^]^ and spike (S) protein, specific for SARS‐CoV‐2. For N and S protein analysis, an anti‐VSV rabbit pAb (Imanis Life Sciences, MN, USA) and an anti‐SARS‐CoV2 (2019‐nCoV) Spike RBD rabbit pAb (Sino Biological, PA, USA) were used diluted 200‐ and 500‐fold, respectively, in Antibody Diluent 2. Both analyses took place with an Anti‐Rabbit Detection Module. For N, 25‐ or 96‐capillary 12–230 kDa Jess/Wes, or Peggy Sue/Sally Sue separation modules were employed. For S protein, the 66–440 kDa size separation modules were used instead. An exposure setting of 4 s was employed to analyze observed peaks. N and S protein concentrations were obtained through a standard curve where necessary.

##### 2.5.1.2 Host Cell protein assay

For host cell protein analysis (HCP), an anti‐VERO goat pAb (Cygnus Technologies, NC, USA), diluted to 5 μg mL^–1^ in Milk Free Antibody Diluent, was used along with an Anti‐Goat Detection Module and 25‐ or 96‐capillary 66–440 kDa Jess/Wes, or Peggy Sue/Sally Sue separation modules. Due to interference in the electropherograms, the fluorescent internal standards were not included in the master mix. The high dynamic range exposure setting was employed to analyze observed peaks. For each analyzed sample, the total peak area, calculated by summing up all detected peak areas, was employed to determine HCP content. HCP concentrations were obtained through a standard curve where necessary. Due to the inclusion of multiple peaks in this assay, instead of a single distinct peak, data analysis also employed the visualization of electropherograms to detect any visible peaks and to consider their associated signal‐to‐noise ratio in samples of interest.

#### 2.5.2 Microplaque infectivity assay

Viral potency measurements were made using an automated and miniaturized plaque assay. This assay employed Vero cells and 96‐well tissue culture microplates (Corning Life Science) and included multiple steps wherein the plates were incubated at room temperature unless specified otherwise. While transitioning from one step to the next, the plates were decanted by aspirating out the contents of their wells. All used chemicals were from Sigma Aldrich unless specified otherwise.

African green monkey kidney Vero cell line was sourced from Merck & Co., Inc., (Kenilworth, NJ, USA) cell banks, grown and maintained in T175 flasks (Corning Life Science), and cultured with Gibco DMEM/High Glucose (Thermo Fisher Scientific Inc.), supplemented with 10% fetal bovine serum (FBS) (ATCC, VA, USA) and 1% Gibco Penicillin‐Streptomycin (Pen‐Strep) (Thermo Fisher Scientific Inc.), in a humidified incubator at 37°C with 5% pCO2. Cells were dissociated using Gibco Trypsin‐EDTA (0.25%), phenol red (Thermo Fisher Scientific Inc.) for 5 min at 37°C, 5% pCO2, > 90% rH, and quenched with Gibco DMEM/High Glucose (10% FBS, 1% Pen Strep). Dissociated cells from multiple flasks were pooled and resuspended in Gibco DMEM/High Glucose (10% FBS, 1% Pen Strep). Cell viability was determined prior to seeding with Cedex automated cell counter and trypan blue (Roche Diagnostics GmbH, Mannheim, Germany).

Vero cells were seeded at 40,000 cells per well in Gibco DMEM/High Glucose (2% FBS, 1% Pen Strep). The plates were incubated overnight at 37°C, 5% pCO2, > 90% rH until 90% confluence. They were then decanted and infected with the addition of a viral inoculum (serially diluted virus) to each of their wells. Post‐infection, the plates were incubated at 37°C, 5% pCO2, > 90% rH for 4 h when an overlay medium was added to their wells. This was comprised of Gibco DMEM/High Glucose (10% FBS, 1% Pen Strep, and 1% Methyl Cellulose (Fisher Scientific, NH, USA)). After its addition, the plates were incubated for an additional 24 h at 37°C, 5% pCO2, > 90% rH. The plates were then emptied and fixed for 30 min with the addition of a 3.7% formaldehyde solution. Post fixation, the formaldehyde solution was replaced with a HyClone Phosphate Buffered Saline (PBS) solution (Cytiva) and 0.5% Triton was added to the plates before they were incubated for 20 min. Following this, the plates were decanted and simultaneously blocked and stained with the addition of a 0.1% Tween‐20, 1% BSA in PBS solution (Teknova, CA, USA) containing 1 μg mL^–1^ Invitrogen Hoechst 33342 (Thermo Fisher Scientific Inc.) and their incubation for 30 min. The plates were then decanted and immunostained with 1 μg mL^–1^ SARS‐CoV‐2 Spike Neutralizing Rabbit Monoclonal Antibody (Sino Biological US Inc., PA, USA). During this step, the plates were incubated for 1 h before they were washed in three cycles with 0.1% Tween‐20 in PBS. A primary SARS‐CoV‐2 antibody, conjugated to a 1:100 diluted Alexa Fluor 488 AffiniPure Donkey Anti‐Rabbit IgG (Jackson Immuno Research, PA, USA), was added to the decanted plates which were then incubated for 1 h. Prior to their imaging, the plates were washed in three cycles with 0.1% Tween‐20 in PBS followed by a final addition of PBS to their wells. Automated image acquisition was completed using an EnSight Multimode plate reader (Perkin Elmer, MA, USA) and the formed plaques were partitioned and counted using Kaleido software (Perkin Elmer). Plaque titer, or infectivity, was calculated using Equation (2).

(2) $$\begin{array}{ccc} Plaque\;Titer & = & \left(Plaque\;Forming\;Units/Inoculum\;Volume\right)\\ & & \times \;Dilution\;Factor \end{array}$$

#### 2.5.3 ELISA host cell protein assay

Residual Vero host cell protein (HCP) was measured using a commercial Cygnus HCP ELISA assay kit (Cygnus technologies, NC, USA), deployed as per the manufacturer's instructions. The concentration of the residual HCP in test samples was determined by interpolating from an HCP standard curve.

#### 2.5.4 Vero host cell DNA qPCR assay

Host cell DNA (hcDNA) was isolated from test samples using a phenol/chloroform‐based extraction method. Here, an in‐house prepared, heterologous internal control was added to each sample along with 1.6 mg mL^–1^ Proteinase K (Thermo Fisher Scientific Inc.), 0.32% sodium dodecyl sulfate (Sigma‐Aldrich), and 0.04 g L^–1^ linear acrylamide (Thermo Fisher Scientific Inc.), followed by incubation at 56°C for 30 min. An equal volume of phenol/chloroform/isoamyl alcohol (25:24:1 solution, buffered to pH ≈8.0; Sigma‐Aldrich) was then added and vigorously mixed by vortexing, followed by centrifugation at 16,000 × *g* for 5 min. The top aqueous layer was transferred to a new tube in which 0.3 M sodium acetate (pH 5.2; Sigma‐Aldrich), 0.02 mg mL^–1^ linear acrylamide, and 1 μL Pellet Paint NF co‐precipitant (MilliporeSigma) were added and mixed. An equal volume of isopropyl alcohol (Sigma‐Aldrich) was added to each tube which was then mixed and centrifuged at 16,000 × *g* for 5 min to pellet the precipitated nucleic acids. The supernatant was removed, and the pellet was washed with the addition of 500 μL cold 70% ethanol (Decon Laboratories, PA, USA), followed by centrifugation at 16,000 × *g* for 2 min. The supernatant was removed, and the pellet was dried in a 37°C oven for approximately 1 h. The dried pellet was resuspended in 160 μL nuclease‐free water (Thermo Fisher Scientific Inc.) until completely dissolved.

Vero hcDNA was measured in the extracted test samples by real‐time quantitative polymerase chain reaction (qPCR), using a specific genomic DNA target, and against a series of five 10‐fold dilutions of Vero genomic DNA reference standard (ATCC CRL‐1587D), ranging from 200 ng mL^–1^ down to 20 pg mL^–1^. Each extracted test sample, standard dilution, and no‐template control (nuclease‐free water) was tested in triplicate wells of an Applied Biosystems MicroAmp Optical 96‐Well Reaction Plate (Thermo Fisher Scientific Inc.) containing qPCR reaction mix. This was comprised of 1X TaqMan Universal PCR Master Mix (Thermo Fisher Scientific Inc.), 400 nM forward primer, 400 nM reverse primer, and 200 nM probe final concentration. qPCR was performed using the ViiA 7 or QuantStudio 7 Flex sequence detection system (Thermo Fisher Scientific Inc.) with the following thermocycling conditions: 50°C for 2 min; 95°C for 10 min; 40 cycles of 95°C for 15 s, 58°C for 1 min. Vero hcDNA concentration in the test samples was determined by interpolation against the reference standard dilution series log‐linear regression. The sequences of the forward and reverse primers, and the probe were 5′‐GAGGCTGAGAAGGGCAGATTAC‐3′, 5′‐TTAGTAGAGACAGGGTTTCACCATGT‐3′, and 5′‐6FAM‐AGGTCAGGAGTTCGAG‐MGB‐3′, respectively. They were prepared at desired stock concentrations in 1X TE buffer, pH 8.0 (Thermo Fisher Scientific Inc.). A separate qPCR assay was performed for the quantification of the heterologous internal control in the extracted test samples to assess nucleic acid recovery.

#### 2.5.5 Transmission electron microscopy

Fresh, unused Capto Core 700 resin was imaged via transmission electron microscopy (TEM) along with used and cleaned in‐place resin. All resin samples were fixed with 4% paraformaldehyde, and 1% glutaraldehyde in 0.1 M phosphate buffer for two hours, washed with 0.1 M phosphate buffer and post‐fixed with 2% osmium tetroxide for one hour. Samples were then washed two times with 0.1 M phosphate buffer, followed by their dehydration in graded anhydrous ethanol in DI water (50%, 70%, 90%, 100%). Next, samples were infiltrated overnight with a 50% (v/v) saturated mixture of LR White Embedding Resin (Electron Microscopy Sciences, PA, USA) and ethanol, followed by two changes of 100% LR White resin and their curing overnight at 65°C for their embedding. The embedded resin samples were ultramicrotomed (100 nm thickness), stained with uranyl acetate and lead citrate, and viewed with a 120 kV FEI Tecnai Spirit Biotwin transmission electron microscope (Thermo Fisher Scientific Inc.). All chemicals were from Sigma Aldrich unless specified otherwise.

#### 2.5.6 SDS‐PAGE

CCCH, fraction pools, and resin extracts were tested via gel electrophoresis using NuPAGE 12% Bis‐Tris 1.0 mm 10–15 lane gels (Invitrogen, CA, USA). For this purpose, 700 μL of denaturing buffer was prepared by mixing 200 μL of NuPAGE Sample Reducing Agent (10X) (Invitrogen) and 500 μL of NuPAGE LDS Sample Buffer (4X) (Invitrogen). For each analysis, 14 μL of denaturing buffer and 26 μL of the sample were mixed in separate wells of a Thermo Scientific Armadillo PCR 96‐well plate which was then sealed with Thermo Scientific Nunc sealing tape and centrifuged for 3 min at 2500 rpm on a Sorvall Legend XTR (Thermo Fisher Scientific Inc.). The PCR plate was then denatured in a Mastercycler Gradient (Eppendorf) for 10 min at 70°C. Following denaturation, up to 25 μL of the sample were loaded into separate lanes of a gel with the latter also including a lane loaded with 5 μL of Mark12 Unstained Standard (Invitrogen). The prepared gels were electrophoresed in a 1X MOPS running buffer, prepared from NuPAGE MOPS SDS Running Buffer (20X) (Invitrogen), for 50 min at 200 V. The gels were then stained with SYPRO™ Ruby Protein (Thermo Fisher Scientific Inc.), according to the manufacturer's microwave protocol, and imaged on a Gel Doc™ EZ System (Bio‐Rad Laboratories, Inc., CA, USA). The images were analyzed using Image Lab Software v6.0.1 (Bio‐Rad Laboratories, Inc.). The location of VSV∆G‐SARS‐CoV‐2 viral product proteins, including large polymerase (L), phosphoprotein (P), and matrix protein (M), in addition to proteins N and S, are denoted in gel images where clearly visible.

### 2.6 Data analysis

The data generated from the analytical methods were employed to make qualitative (i.e., SDS‐PAGE and TEM images) and quantitative (i.e., chromatograms, product yields, and impurity contents) assessments. For the latter, data analysis is based on inspection of trends, by plotting and processing chromatograms, and one‐way analysis of variance, with pairwise comparisons using Tukey's method to control Type I errors,^[^ ^21^ ^]^ using the resin re‐use cycle number as the independent variable. In the absence of an estimate of pure error from technical and analytical replicates, the non‐parametric Spearman's rank correlation coefficient,^[^ ^22^ ^]^ *ρ*, was employed to determine the presence of a relationship between assay results and resin re‐use cycle number. The aforementioned processing of chromatograms involves the aggregation of measurements in total signals for each phase of a chromatography run. For HT scale chromatograms, the measured absorbances of the collected fractions were corrected via subtraction against a corresponding blank (i.e., mobile phase) and then normalized over their pathlength (negative values were replaced by zero). These processed absorbances were then summed over the fractions collected during each phase to yield the total signals (Au/cm) corresponding to the loading, washing, CIP, and post‐CIP flushing of a column (i.e., FT, Wash, CIP1, CIP2, CIP3, and PCIP, respectively). For lab scale chromatograms, the total signals (AuÍmL) were estimated by integrating the measured absorbances from each of the aforementioned six phases. For impurity data, log reduction values were estimated using the base 10 logarithm of the ratio between the starting and final impurity levels. This accounted for volumetric concentration factors where applicable.

## 3 RESULTS AND DISCUSSION

### 3.1 High throughput screening of CIP agents

**FIGURE 2:**
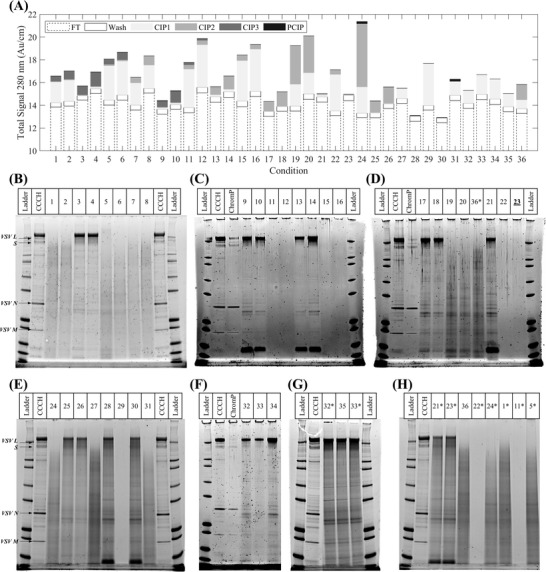
Evaluation of 36 cleaning in place conditions, implemented by applying up to three different cleaning agents in three sequential steps, each deployed at the end of a purification run to generate the chromatography flowthrough product pool (ChromP) from clarified cell culture harvest (CCCH) using RoboColumns: (A) Total signals from optical absorbance measurements at 280 nm from fractions collected during the loading, washing, application of cleaning in place steps 1–3, and post‐CIP flushing the columns (FT, Wash, CIP1, CIP2, CIP3, and PCIP, respectively) per cleaning condition; (B–H) SDS‐PAGE analysis of resin extracts collected at the end of each CCCH purification and deployment of the cleaning conditions. In (B–H), boxes above each gel denote the samples tested per lane and gel. In these boxes, numbers correspond to cleaning conditions, also shown on the x‐axis of (A), and asterisks denote replicated experiments using previously tested cleaning conditions. In (D), the underlined condition 23 is a spurious result due to an error in loading the gel

Capto Core 700 is a multimodal resin with a highly hydrophobic ligand. Recommendations for CIP this resin are provided in the manufacturer's instructions. However, these require flammable solvents, such as isopropanol and n‐propanol. The use of such solvents can become limiting at the pilot plant and commercial scales due to OSHA regulations requiring handling to take place within an explosion‐proof facility.^[^ ^23^, ^24^ ^]^ To overcome this, the screening of the cleaning agents at the HT scale sought to identify alternatives by testing 36 conditions, each designed to deploy up to three cleaning agents per condition in three sequential steps (CIP1, CIP2, and CIP3) (Table S1, Supporting Information). For each test, columns were used to purify CCCH and the chromatographic traces were converted into total signals (Figure 2A, Supporting Information). The total signal estimation for conditions 1–8 differed from the one described in Section 2.6 since it was based on pathlength normalized absorbances alone. Moreover, at the end of each RoboColumn run, the resins, each corresponding to a different cleaning condition, were extracted and analyzed by SDS‐PAGE (Figure 2B–H).

The FT and Wash total signals remained consistent across the 36 separations, with averages of 13.87 ± 0.69 Au cm^–1^ and 0.44 ± 0.03 Au cm^–1^, respectively, (Figure 2A) supporting the reproducibility of the purification of the VSV∆G‐SARS‐CoV‐2 vaccine candidate across all 36 runs. A low PCIP total signal was obtained across all tested conditions (average of 0.02 ± 0.04 Au cm^–1^ from Figure 2A) since this step was added to facilitate the resin extraction by storing it in a neutral pH Tris buffer. Hence, flushing the columns post‐CIP had no contribution to their cleaning. Similarly, CIP3, applied for conditions 1–6 and 9–12 (Table S1, Supporting Information), led to the lowest signal amongst CIP1–CIP3 except for conditions 3, 4, 9, and 10 (Figure 2A). This suggested that the application of CIP3 could be beneficial in cleaning the resin by removing any additional residuals present after the application of CIP1 and CIP2. Such behavior was not supported, however, by the analysis of the resin extracts from conditions 1–6 (Figure 2B) and 9–12 (Figure 2C) demonstrating that only conditions 11 and 12 led to extracts free of residuals (Figure 2C). For these two conditions, CIP1 included a mixture of 30% n‐propanol/1 N NaOH and was found to be the only cleaning agent leading to resin extracts free of residuals when deployed during CIP1 in either a two‐step cleaning condition (conditions 15, 16, and 22 in Table S1, Supporting Information, with resin extracts shown in Figure 2C and D, respectively) or when deployed alone (condition 29 in Table S1, Supporting Information, with resin extract shown in Figure 2E).

Conditions including the 30% n‐propanol/1 N NaOH mixture in CIP1 led to both high CIP1 and overall signals (Figure 2A). This agreement between the overall absorbance signal and the absence of residuals in the resin extracts could support the employment of chromatograms as a screening tool to identify conditions with a high likelihood of leading to residual‐free resin extracts. However, conditions including a 30% isopropanol/1 N NaOH mixture in CIP1 (5, 6, 19, 20, 24, and 31 in Table S1, Supporting Information) also led to high overall signals (Figure 2A) while the corresponding resin extracts were neither free of residuals (Figure 2B, D and E) nor considerably cleaner from extracts obtained from alternative conditions which had a considerably lower overall absorbance signal (e.g., conditions 1, 2, 7 and 8 vs. 5 and 6 in Table S1, Supporting Information, and Figure 2A and B). Hence, the use of chromatograms to assess cleaning conditions could potentially lead to erroneous conclusions. Here, it is important to highlight that while both high content n‐propanol and isopropanol mixtures with 1 N NaOH are recommended in the manufacturer's instructions as cleaning solutions for Capto Core 700, only the former was found to be effective while the latter was similar in performance to, for example, condition 36 including a 1 N NaOH cleaning agent in CIP1 (condition 24 vs. 36 in Table S1, Supporting Information and Figure 2H). Hence, the use of the isopropanol and NaOH solution mixture represented a sub‐optimal cleaning agent. The presence of NaOH was determined to be a necessary component of the 30% n‐propanol/1 N NaOH mixture since in its absence (condition 28 in Table S1, Supporting Information) the returned resin extract was not clean (condition 29 vs. 28 in Figure 2E). The screening experiments identified therefore a single cleaning agent capable of cleaning Capto Core 700 resin used to purify CCCH. The use of n‐propanol and NaOH was characterized further to determine a cleaning strategy for the resin.

#### 3.1.1 Cleaning in place strategy

While screening experiments did not lead to a flammable solvent‐free cleaning condition, a potential clean‐in‐place strategy for Capto Core 700 resin, capable of meeting regulatory requirements, was identified: (1) After its loading and washing, the column would be flushed with 0.5 N NaOH for a total of 18 min; (2) The column would then be cleaned in place by flushing it with an n‐propanol/NaOH solution for a sufficiently long contact time, and finally (3) The column would be flushed with 0.5 N NaOH for a total of 18 min. Here, the first step represents a viral inactivation and sanitization step, and the last one is a storage step. This strategy would allow the shipment of a used column, after it was inactivated, to a facility, meeting regulatory requirements for handling flammable solvents, to be cleaned in place. Then the column would be stored in 0.5 N NaOH before it was shipped back to continue processing.

**FIGURE 3:**
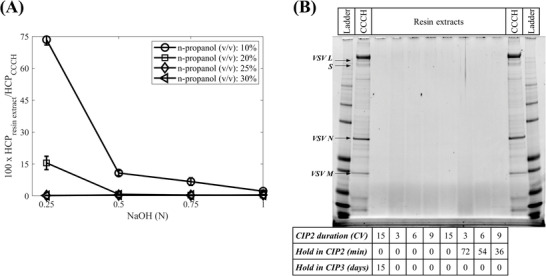
Investigation of factors affecting the n‐propanol/NaOH cleaning in place strategy using RoboColumns by purifying clarified cell culture harvest (CCCH) and analyzing the generated resin extracts: (A) Relative host cell protein (HCP) content in Capto Core 700 resin extracts compared to HCP content in CCCH as a function of n‐propanol and NaOH concentrations in cleaning in place solution; (B) SDS‐PAGE analysis of the resin extracts analyzed in (A). In (B), the top box denotes the samples analyzed and the bottom box denotes the conditions leading to the corresponding analyzed resin extracts

Additional screening was completed with RoboColumns to understand the impact of n‐propanol and NaOH concentrations and the duration of the second step (CIP2) on the cleaning of the Capto Core 700 resin (Figure 3). The duration studies expanded only on the second step while maintaining other steps of the CIP strategy (i.e., CIP1 and CIP3). This was implemented based on the identification of the n‐propanol and NaOH solution mixture as the only agent capable of cleaning the Capto Core 700 resin (Figure 2). The concentrations of the two components in the CIP2 solution were both found to be significant and to affect the content of residuals in the resin extracts in a non‐linear fashion, which included quadratic and interaction terms as determined by regression analysis (Figure 3A). Here, cleaning the Capto Core 700 resin, previously used to purify CCCH, with NaOH and n‐propanol concentrations greater than 0.75 N NaOH and 20% in the CIP2 solution, respectively, led to a radical reduction of adsorbed HCP presence in the resin extracts. These tests were expanded by the application of these concentrations to clean in place Capto Core 700 columns exposed to CCCH of additional viral products produced in adherent Vero cells which presented a higher impurity content to the resin than the one observed for the VSV∆G‐SARS‐CoV‐2 vaccine candidate. This revealed that the concentrations of n‐propanol and NaOH in their mixture needed to be further increased in tandem to ensure the removal of fouling residuals from the Capto Core 700 resin (data not shown). Hence, NaOH concentrations of >0.75 N at an n‐propanol concentration of 30% were determined to be desirable.

These concentrations were employed while investigating the impact of the duration of CIP2 under two modes, flow or flow and hold. In the former case, the resin was left in contact with the CIP2 solution for 3, 6, 9, and 15 CVs under flow, with a residence time of 6 min, and then the columns were stored after flushing them with 0.5 N NaOH in CIP3. In the latter case, the resin was contacted with the CIP2 solution for the same durations under flow, but an additional hold time was applied to a total CIP2 time of 90 min before the columns were flushed with 0.5 N NaOH in CIP3. For example, a column contacted for 3 CVs (i.e., 18 min) with the 30% n‐propanol/0.75 N NaOH solution under flow was held for an additional 72 min in CIP2, whereas a column contacted for 9 CVs (i.e., 54 min) with the solution under flow was held for an additional 36 min in CIP2 before CIP3. Here, the addition of a hold in CIP2 was tested to explore the possibility of minimizing the consumption of the 30% n‐propanol/0.75 N NaOH solution by evaluating if a Capto Core 700 column could be cleaned by contacting the resin with this solution during a hold and without flowing fresh solution through it. The HCP analysis of the resin extracts from these experiments, with the quantitative western blotting assay, yielded HCP contents <LOQ for each condition. This supported the selection of the 30% n‐propanol and 0.75 N NaOH composition of the CIP2 solution in these CIP2 duration studies. Further testing with SDS‐PAGE analysis (Figure 3B) corroborated the results of the quantitative assay, but here, the conditions wherein CIP2 was applied for 3 CVs under flow, with or without a hold, led to darker lane images compared to the rest of the tested conditions. This suggested that the application of CIP2 for short periods was not entirely successful in removing residuals completely from used Capto Core 700 resin. For CIP2 applications of greater than 3 CVs, the presence of a hold appeared to lead to marginally cleaner lanes (Figure 3B) whereas the application of CIP2 for 15 CVs combined with resin storage in CIP3 for 15 days did not lead to any additional removal of foulants from the resin (Figure 3B). These suggested that given an effective cleaning condition, the use of a hold would not add to its efficiency.

While these results demonstrated that a 30% n‐propanol/0.75 N NaOH solution could be employed in cleaning a Capto Core 700 column used for one purification of the VSV∆G‐SARS‐CoV‐2 vaccine candidate, a decision was made to adopt a more aggressive CIP2 solution (i.e., 30% n‐propanol/1 N NaOH) and a contact time under flow of 30 min for multi‐cycle resin re‐use experiments. This aimed to increase the likelihood of a successful CIP strategy in multiple resin re‐use cycles with a more conservative approach.

### 3.2 High throughput resin re‐use experiments

**FIGURE 4:**
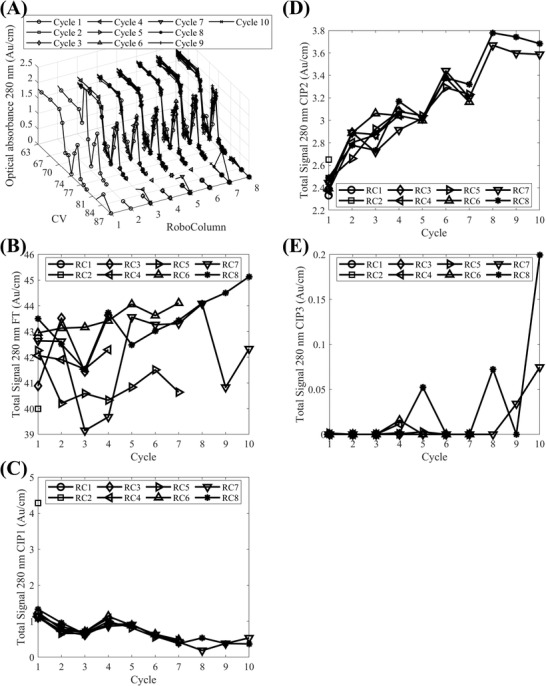
Evaluation of the impact of the three‐step cleaning in place strategy at the high throughput scale across ten resin re‐use cycles based on chromatograms: (A) Blank and pathlength corrected optical absorbance at 280 nm per RoboColumn (RC) plotted against column volumes (CVs) across the ten re‐use cycles; (B–E) Total signals form optical absorbance measurements at 280 nm from fractions collected during the loading of the columns (FT) and the application of cleaning in place steps 1–3 (CIP1–CIP3, respectively), respectively, per RC as a function of resin re‐use cycle. In (A) the y‐axis represents the RoboColumn number and lines with markers (○), (□), (◊), (⊲), (⊳), (△), (▽), (⋆), (+), (×) correspond to resin re‐use cycles 1–10, respectively. In (B–E) lines with markers (○), (□), (◊), (⊲), (⊳), (△), (▽), (⋆) correspond to RCs 1–8, respectively. In (A) the chromatograms are zoomed in by plotting the data from the 63^rd^ column volume onwards

The formulated CIP strategy was deployed in HT resin re‐use experiments where 8 RoboColumns (i.e., RC1–RC8) were employed to purify the VSV∆G‐SARS‐CoV‐2 vaccine candidate across 10 cycles (Figure 1A). Chromatographic traces (Figure 4A and Figure S2, Supporting Information) were converted to total absorbance signals for the column loading, and CIP1‐CIP3 application phases (Figures 4B–E, respectively), for each RoboColumn and cycle, by summing blank and pathlength corrected absorbance measurements at 280 nm from the collected fractions. Only RC6 and RC8 displayed a significant positive correlation between the total signal of the flowthrough fractions and the cycle number (Table S2, Supporting Information) indicating an increasing breakthrough with increasing cycle number. However, the relative increase in the total signal in the last cycle, compared to the first one, was low (≈2% and ≈3% for RC6 and RC8, respectively). Moreover, such significant positive correlations were not observed for columns used for the same number of cycles to RC6 and RC8 (e.g., RC5 and RC7, respectively, in Table S2, Supporting Information).

**FIGURE 5:**
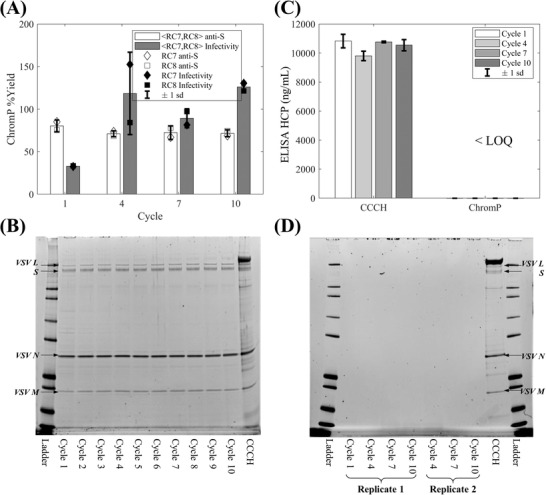
Evaluation of the impact of cleaning in place strategy across ten resin re‐use cycles at the high throughput scale based on product yield and purity analysis and resin extract analysis: (A) Bar plot of chromatography flowthrough product pool (ChromP) yields for resin re‐use cycles 1, 4, 7, and 10, averaged across RoboColumns (RCs) 7 and 8, based on anti‐Spike (S) protein quantitative western blotting and infectivity data; (B) SDS‐PAGE analysis of clarified cell culture harvest (CCCH) and ChromP from RC8 for each resin re‐use cycle; (C) Bar plot of ELISA host cell protein (HCP) analysis results of CCCH and ChromP from RC8 for resin re‐use cycles 1, 4, 7 and 10; (D) SDS‐PAGE analysis of resin extracts obtained post‐cleaning in place the resin from RC2, RC4, RC6, and RC8 at the end of cycles 1, 4, 7 and 10, respectively. In (A) the error bars correspond to ± 1 standard deviation (sd) using the RC7 and RC8 yield data, shown by open (◊) and (□) symbols, respectively, for the anti‐S data, and closed (⧫) and (■) symbols, respectively, for the infectivity data. In (B) RC8 derived flowthrough product pools are shown since it was used across all ten resin re‐use cycles. In (D) the extracts from cycles 4, 7, and 10 were analyzed in duplicate

Across all RoboColumns and cycles, the total signals corresponding to CIP1, CIP2, and CIP3 (Figures 4C–E, respectively) were the highest for CIP2 followed by CIP1 and then CIP3. This agreed with previous observations that the application of CIP2 (30% n‐propanol/1 N NaOH) contributes the most to the cleaning of the resin and the application of CIP3 functions predominately as a storage solution (Figure 2A). Trends between the total signals and the cycle number were observed for CIP1 (Figure 4C) and CIP2 (Figure 4D). For the former, strong negative correlations were in place for RC7 and RC8 whereas, for the latter, RC5–RC8 displayed strong positive correlations (Table S2, Supporting Information). The increasing signals of CIP2 with the cycle number indicate a regime wherein foulants accumulate on the resin, even when it is cleaned in place, which are removed from the resin with its repeated exposure to the CIP strategy. To assess the impact of such accumulation on the yield and purity of the chromatography flowthrough product pool, quantitative product and impurity‐based analytical testing was performed for the flowthrough product pools from RC7 and RC8 (Figure 5A–C).

Chromatography flowthrough product pool yields, based on anti‐S quantitative western blotting, (Figure 5A) were estimated to be on average 73.80% ± 6.02% across cycles 1, 4, 7, and 10 for RC7 and RC8, and the obtained data returned an insignificant effect of cycle number on yields, as determined by one‐way ANOVA (*p*‐value = 0.4561, Table S3, Supporting Information). Here, it is noted that low amounts of the VSV∆G‐SARS‐CoV‐2 vaccine candidate bound to the resin despite its diameter (≈70 nm) being greater than the diameter of the Capto Core 700 resin's pores.^[^ ^25^ ^]^ Likewise, an insignificant impact of the cycle number on the product yields was also determined based on the infectivity data (*p*‐value = 0.0632, Table S3, Supporting Information) (Figure 5A) which led to an average yield of 91.63% ± 43.41% across the four considered cycles. Here, the high variability of the average yield was due to the individual yields from cycle 1 which were considerably lower than those from cycles 4, 7, and 10 (i.e., 32.94% ± 0.56% vs. > 85% in Figure 5A). This was attributed to a lack of cryoprotectant addition to the cycle 1 flowthrough product pool aliquots before they were frozen for their analysis with the infectivity assay. This can lead to a loss of infectivity in the stored samples due to their freezing and thawing prior to their testing. Excluding the cycle 1 chromatography product yield data led to an even lower *F* statistic and hence did not change the derived conclusions (Table S3, Supporting Information).

Higher‐resolution chromatography flowthrough product pool yield results were obtained based on the anti‐N quantitative western blotting data since this assay was deployed for each RoboColumn across the 10 cycles (Figure S3 and Table S4, Supporting Information). These data indicated a significant difference in the product yields for RC8 (*p*‐value = 0.0066, Table S4, Supporting Information); a difference was observed between cycle 3 and cycles 4 and 10 (i.e., 61.32% ± 8.45% vs. 90.74% ± 10.51% and 96.67% ± 16.10%, respectively) (Table S5, Supporting Information). Conversely, for RC7, which also went through all 10 cycles of resin re‐use (Figure 1A), no significant differences were detected (Table S4, Supporting Information). This, along with the fact that for RC8 only 2 out of 45 pairwise comparisons (Table S5, Supporting Information) were shown to be significantly different from each other, and none of the pairwise comparisons between cycle 1 and later cycles were statistically significant, led to the conclusion that these results corroborated the infectivity and anti‐S quantitative western blotting yield data. Hence, these data support that Capto Core 700 chromatography flowthrough product pool yields are not dependent on the number of resin re‐uses.

The absence of persistent foulants, accumulating from one re‐use cycle to the next, was also indicated by the analysis of the product pools (Figure 5B and C) and resin extracts (Figure 5D) for impurity presence. The chromatography flowthrough product pools across all cycles displayed an identical band pattern and purity based on SDS‐PAGE (Figure 5B) and the ELISA HCP assay, for cycles 1, 4, 7, and 10, showed that the chromatography step reduced considerably and consistently the HCP content in CCCH to < LOQ in the product pools (Figure 5C). The deployment of the higher throughput HCP quantitative western blotting assay agreed with these results since the product pools across all cycles for RC7 were also determined to be < LOQ. Similarly, hcDNA content obtained based on qPCR analysis of product pools from cycles 1, 4, 7, and 10, for both RC7 and RC8, was also found to be < LOQ. The analysis of resin extracts via SDS‐PAGE also evidenced the lack of any residual solutes post the application of CIP (Figure 5D). This was also confirmed by HCP via quantitative western blotting analysis of the resin extracts (data not shown). Here, it is important to highlight that the preparation of the resin extracts represents a ≈17‐fold volumetric concentration which would increase the chances of observing a quantifiable level of HCPs in the presence of accumulation of foulants due to incomplete CIP of the resin. Hence, the lack of quantifiable solutes in such samples, tested with sensitive assays, strengthens the positive evaluation of the designed CIP strategy despite the chromatogram‐based trends observed in Figure 4C and D.

### 3.3 Lab‐scale resin re‐use experiments

Following the successful demonstration of the three‐step CIP strategy at the RoboColumn scale, lab‐scale resin re‐use experiments were performed using a 20 mL pre‐packed Capto Core 700 column. This served to verify the effectiveness of the CIP strategy for 10 re‐use cycles and provided enough product to perform the post‐Capto Core 700 UF/DF step.

**FIGURE 6:**
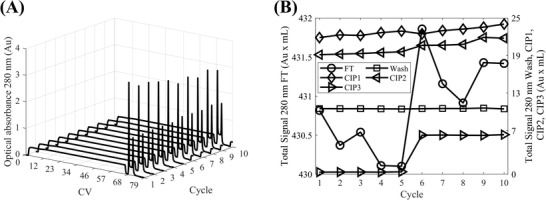
Evaluation of the impact of cleaning in place strategy at the lab scale across ten resin re‐use cycles based on chromatograms: (A) Optical absorbance at 280 nm plotted against column volumes (CVs) across the ten re‐use cycles; (B) Total signal from integrated optical absorbances at 280 nm from effluent collected during the loading (FT) and washing (Wash) of the column, and the application of cleaning in place steps 1–3 (CIP1–CIP3, respectively) to the column as a function of column re‐use cycle. In (B), the FT Total signal is on the left‐hand side y‐axis whereas the Wash and CIP1–CIP3 Total signals are on the right‐hand side y‐axis

Similar to the HT scale results (Figure 4A), the recorded chromatographic traces from the lab‐scale runs (Figure 6A) showed a near‐perfect overlap across the cycles. This was especially true during the loading (Figure 6B) and washing (Figure 6B) of the column wherein the integrated chromatograms from these two phases led to signals that were independent of the column re‐use cycle. For the former, the flowthrough peak showed an increase from cycles 1–5 to cycles 6–10, which was < 1% and hence insignificant. A step increase between these two sets of cycles was also observed in the recorded signal for CIP3 whereas within each set the CIP3 signal remained virtually constant (Figure 6B). For cycles 6–10, the increase in the CIP3 signal is observed in the recorded chromatograms (Figure 6A); it increased ≈1 CV after the completion of CIP2 and remained constant until the completion of CIP3. Hence, such an increase represents a baseline shift from CIP2 to CIP3 instead of indicating the elution of solutes. This behavior was attributed to an error in the preparation of the CIP3 solution as no other sources of error could be identified.

Conversely, for CIP1 and CIP2 (Figure 6B) a strong positive correlation was observed between the integrated signals and the number of re‐use cycles (*ρ* = 0.95, *p*‐value < 0.0001 and *ρ* = 0.99, *p*‐value < 0.0001, respectively). Contrary to the HT scale data, here the CIP2 step did not lead to the highest observed signal amongst CIP1–CIP3 (Figure 6B). This was attributed to the fact that at the HT scale the recorded signals were blank corrected whereas this was not the case for the lab‐scale data. Nevertheless, the existence of strong positive correlations for CIP1 and CIP2 for the lab‐scale data could indicate an incomplete cleaning of the resin, also considered for the HT scale resin re‐use study (Figure 4D). This was sought to be verified by the analysis of chromatography flow through product pools, resin extracts, and intermittent BSA DBC measurements (Figure 1B).

**FIGURE 7:**
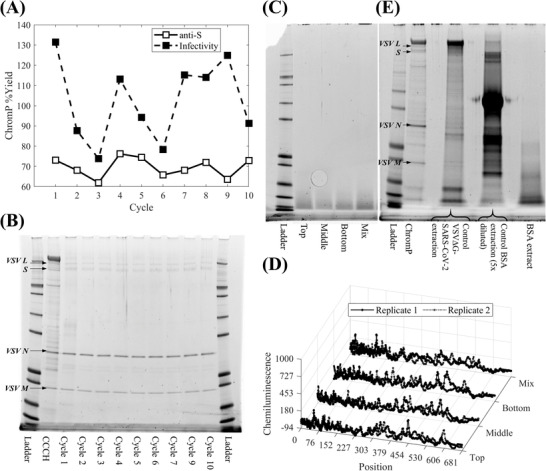
Evaluation of the impact of cleaning in place strategy across ten resin re‐use cycles at the lab scale based on product yield and purity analysis and resin extract analysis: (A) Chromatography flowthrough product pool (ChromP) yields per cycle based on anti‐Spike (S) protein quantitative western blotting (□) and infectivity data (■); (B) SDS‐PAGE analysis of clarified cell culture harvest (CCCH) and ChromP per cycle (cycle 8 was excluded); (C) SDS‐PAGE analysis of resin extracts from resin samples collected at the end of the ten resin re‐use cycles from the top third of the bed volume (Top), the middle third of the bed volume (Middle), the bottom third of the bed volume (Bottom) and their mix (Mix); (D) Electropherograms from HCP quantitative western blotting analysis of resin extracts from the Top, Middle, Bottom, and Mix column resin samples; (E) SDS‐PAGE analysis of ChromP and control VSV∆G‐SARS‐CoV‐2 and BSA resin extracts. In (D) markers (○) and (□) denote analytical replicates and the electropherograms indicate the absence of HCPs in the tested extracts

Product yields, based on anti‐S quantitative western blotting and infectivity data (Figure 7A), were determined to be on average 69.60% ± 4.83% and 102.40% ± 19.91%, respectively, which were close to those obtained from the HT resin re‐use experiments (i.e., 73.80% ± 6.02% and 91.63% ± 43.41%, respectively). The higher variability observed for the infectivity data‐based yields is attributed to the nature of the assay itself and not to the variability of the chromatography step. The product yields per cycle from both assays were also found to be independent of the cycle number in both cases (*ρ* = –0.12, *p*‐value = 0.7588 and *ρ* = 0.13, *p*‐value = 0.7329, respectively). At the same time, the ELISA HCP and hcDNA results from the chromatography product for each cycle were < LOQ, a result also corroborated for the former by the SDS‐PAGE analysis of the flowthrough product pools (Figure 7B).

While these results suggested the success of the CIP strategy, additional rigor was applied in evaluating its effectiveness. For this purpose, BSA *DBC_10%_* measurements were made between cycles 5 and 6 (*DBC_10%_* = 10.55 g L^–1^) and after cycle 10 (*DBC_10%_* = 10.43 g L^–1^) (Figure 1B). The estimated *DBC_10%_* values were close to each other and to the reference, *DBC_10%_* (11.34 ± 0.10 g L^–1^), determined using three fresh 20 mL columns. In comparison, when a fourth fresh 20 mL column was used to purify 245 CVs of CCCH and to then determine BSA's *DBC_10%_*, without being cleaned in place, the returned *DBC_10%_* was estimated to be 4.66 g L^–1^. This significant reduction demonstrates the extent of the impact of an ineffective CIP of a CCCH exposed Capto Core 700 column. Hence, while the BSA *DBC_10%_* values obtained during the re‐use experiments were marginally lower than the reference one, the difference was deemed to be insignificant and the CIP strategy was further concluded to be effective.

To further assess the performance of the CIP strategy for the lab‐scale experiments, resin samples were collected from the top, middle, and bottom layers of the employed 20 mL column, (Figure 1B) and analyzed individually and in their combination. Here, their SDS‐PAGE analysis (Figure 7C) showed the presence of a small amount of residuals, which did not vary significantly from top to bottom layer. While this was different from the results observed from the HT scale (Figure 5D), HCP analysis via quantitative western blotting returned, for both scales, an HCP content below LOQ in the resin extracts, as depicted by electropherograms containing solely background signal (Figure 7D). Similarly, representative TEM images compared fresh, unused Capto Core 700 resin (Figure S4A, Supporting Information) to the aforementioned three resin samples (Figures S4B–D, Supporting Information) from the cycled 20 mL Capto Core 700 column. Here, the light gray areas depict the embedding material whereas darker grey areas depict the resin backbone. The structure of the Capto Core 700 resin was confirmed to be comprised of a fibrous mesh with large pores (Figure S4A, Supporting Information), as observed previously.^[^ ^25^ ^]^ The images of the three resin samples from the re‐used 20 mL Capto Core 700 column (Figures S4B–D, Supporting Information) were identical to each other and to the image from the fresh resin sample (Figure S4A). Hence, the structure of the resin itself was not affected by the re‐use of the column. Moreover, previously published TEM images of used but not cleaned Capto Core 700 resin typically depict foulants as large dark globules.^[^ ^25^ ^]^ Based on this, the absence of such globules in the TEM images in Figures S4B–D, Supporting Information, indicated a lack of significant amounts of foulants in resin samples from the re‐used and cleaned in place Capto Core 700 column. The same applied when images were taken near the edge of the resin's beads, as opposed to their interior as shown in Figure S4, Supporting Information, since no deposition of a dark front was observed on their surface (data not shown).

The presence of protein content in the resin extracts of the 20 mL Capto Core 700 column was investigated further via the execution of control experiments at the RoboColumn scale. These generated extracts were derived from the purification of the VSV∆G‐SARS‐CoV‐2 vaccine candidate from CCCH and from scaling down the lab‐scale BSA DBC measurements. For these two tests, upon completion of column loading and washing, the resin was extracted prior to its cleaning with the CIP strategy to generate control extracts depicting the extent of the presence of residuals in uncleaned Capto Core 700 columns (i.e., control VSV∆G‐SARS‐CoV‐2 and control BSA extraction lanes in Figure 7E). A third control experiment at the RoboColumn scale was conducted to simulate the BSA DBC measurements but here the column was cleaned before the resin was extracted (i.e., BSA extract in Figure 7E). These analyses confirmed the significant presence of bound HCPs and BSA to Capto Core 700. More importantly, the CIP strategy resulted in the presence of BSA‐related peptides in the cleaned Capto Core 700 resin even if it was considerably reduced compared to the control BSA extract and to such an extent that they were not observed in the TEM images (Figure S4, Supporting Information). The presence of BSA‐related residuals was also observed via SDS‐PAGE when employing a modified CIP strategy which used 30% isopropanol/1 N NaOH in CIP2 (Figure S5A, Supporting Information). The sub‐optimal performance of the CIP strategy in removing BSA from Capto Core 700 (Figure 7E), led to the conclusion that the residuals in the lab‐scale extracts (Figure 7C) were comprised of BSA‐related peptides, introduced to the column during the BSA DBC measurements, and not of HCPs, introduced to the column from the purification of the VSV∆G‐SARS‐CoV‐2 vaccine candidate from CCCH. This also took into consideration the analyses of the flowthrough product pools from cycles 1–10 of the lab‐scale multi‐cycle resin re‐use experiments (Figure 7A,B), the scalability of the HT scale multi‐cycle experiments (Figure 5A,B), and the absence of foulants in their resin extracts (Figure 5D).

Here, it needs to be highlighted that the inefficiency of the CIP strategy in cleaning BSA from the Capto Core 700 column was attributed to the duration of the CIP2 step. The HT scale experiments showed that the BSA‐loaded RoboColumns were flushed with CIP3 (i.e., 0.5 N NaOH) before the entire collection of the CIP2 peak (Figure S5B, Supporting Information). This was supported by the virtually equal two BSA *DBC_10%_* measurements made during the lab‐scale resin re‐use experiments (i.e., 10.55 and 10.43 g L^–1^ measured after cycles 5 and 10, respectively); residuals remaining bound to the column post its cleaning would lead to a reduction in the second *DBC_10%_* measurement. Instead, the continued application of the CIP strategy, at the end of each cycle, acted cumulatively and led to the clearance of such residuals and an unchanged BSA *DBC_10%_* value. This cumulative cleaning effect was not observed via resin extraction since it occurred after the 10 purification cycles. There, the 20 mL Capto Core 700 column was loaded with BSA anew, for its second DBC measurement, and then cleaned in place one last time before its resin was collected, extracted, and analyzed (Figure 1B). This effectively reset the state of the BSA‐related residuals in the column and led to the trends observed in Figure 7C.

#### 3.3.1 Impact of resin re‐use on the final purified product

**FIGURE 8:**
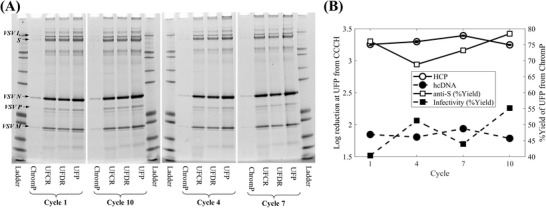
Evaluation of the impact of cleaning in place strategy on the purity and yield of the final purified product (UFP) obtained by processing the chromatography flowthrough product pool (ChromP) through ultrafiltration/diafiltration for lab‐scale resin re‐use cycles 1, 4, 7 and 10: (A) SDS‐PAGE analysis of clarified cell culture harvest (CCCH), ChromP, concentrated ChromP (UFCR), diafiltered ChromP (UFDR) and UFP; (B) In left‐hand side y‐axis, log reduction of host cell protein (HCP) (○) and DNA (hcDNA) (●) impurities at UFP from CCCH obtained by ELISA and qPCR analysis, respectively. On the right‐hand side y‐axis, step yield at UFP from ChromP based on anti‐Spike (S) protein quantitative western blotting (□) and infectivity data (■). In (B), the HCP log reduction data error bars correspond to ± 1 standard deviation (sd) from analytical replicates

Upon completion of the lab‐scale resin re‐use experiments, the chromatography products from cycles 1, 4, 7, and 10 were thawed and processed further through the UF/DF step (Figure 1B) and process intermediates from the concentration (UFCR) and diafiltration (UFDR) steps were sampled and analyzed along with the final product (UFP). Comparing the UF/DF intermediates and final product from cycle 1 to those of cycles 4, 7, and 10 showed nearly identical band patterns in SDS‐PAGE analysis (Figure 8A). These results were further corroborated by ELISA HCP analysis since the final product returned an average log reduction of HCP content from CCCH of 3.30 ± 0.07 across the four cycles (Figure 8B). Despite the low variability of the average HCP log reduction values, the ELISA HCP data indicated a significant difference between the cycles (*p*‐value = 0.0009 in Table S6, Supporting Information); a higher log reduction was achieved in cycle 7 compared to cycles 1, 4, and 10 (Figure 8B and Table S7, Supporting Information). The analysis of variance results, however, was driven by a considerably low pure error since the returned HCP concentrations from the four cycles had a variability of ≈1.5%–3%. Hence, the increase in log reduction for cycle 7, compared to cycles 1, 4, and 10, corresponded to a difference of < ≈4.5% and combined with the fact that for cycle 7 the concentration factor overshot the target by ≈60% led to attribute this difference to acceptable process variability instead of the re‐use of the column. This was also indicated by observing the similarity of the hcDNA log reduction trends (Figure 8B) to those from the HCP data since cycle 7 was also shown to lead to the highest log reduction compared to the rest of the four cycles. Here, the achieved hcDNA log reduction across the four cycles (1.84 ± 0.07) was lower than the one for HCP.

The SDS‐PAGE analysis of the UF/DF intermediates and final product also indicated similarity of product yields for UFP for cycles 1, 4, 7, and 10 (Figure 8A). This was supported by anti‐S quantitative western blotting and infectivity data returning average UFP step yields from the chromatography flowthrough product pool, across the four cycles, of 74.16% ± 4.13% and 47.70% ± 6.75%, respectively (Figure 8B). For both assays, an insignificant correlation was obtained between the yields and cycle number (*ρ* = 0.40, *p*‐value *=* 0.7500 and *ρ* = 0.80, *p*‐value = 0.3333, respectively). Based on these results, and taking into consideration the sources of inherent process variability, it was determined that the re‐use of the column, with the application of the CIP strategy, enabled the robust production of the VSV∆G‐SARS‐CoV‐2 vaccine candidate for 10 batches.

### 3.4 Assessment of the high throughput approach to establishing a cleaning‐in‐place strategy

The HT scale experiments employed RoboColumns to identify and then deploy cleaning agents in column re‐use experiments. The screening of cleaning agents evidenced the necessity for the simultaneous action of NaOH and n‐propanol to clean the octylamine ligand of the Capto Core 700 resin, with or without the presence of additional cleaning agents (Figure 2C–E). The performed screens also highlighted the importance of confirming the manufacturer's instructions since a 30% isopropanol/1 N NaOH solution was found to be ineffective in all tested cleaning conditions, comprised of both single and multiple cleaning steps (Figure 2B,D–F). The 36 tested conditions also sought to evaluate cleaning agents on a standalone basis and as part of combinations as implemented by their deployment in sequential CIP steps (conditions 27–36 and 1–26, respectively, in Table S1, Supporting Information). This demonstrated a lack of a synergistic action between cleaning agents. For example, the use of 0.5 M acetic acid alone led to a high amount of residuals bound to the column post‐cleaning (condition 32 in Figure 2F). Conversely, condition 1, which employed both 0.5 M acetic acid and 1 N NaOH led to a cleaner resin extract (Figure 2B), which was, however, nearly identical to cleaning condition 36 which employed 1 N NaOH alone (Figure 2H). The lack of such synergistic action for the evaluated cleaning agents, the inability of high concentration acids and chaotropes (condition 35, 34 in Table S1 and Figures 2G, 2F, respectively) to produce clean resin extracts, and time constraints led to the decision to focus on evaluating the deployment of the 30% n‐propanol/1 N NaOH solution in a cleaning strategy instead of extending screens of cleaning agents.

The screening experiments also demonstrated the limitations of evaluating cleaning approaches solely based on chromatographic absorbance traces; conditions leading to high absorbance signals were not always those returning the cleanest resin extracts (e.g., conditions 19 and 20 in Figure 2A and D). Such false positives were avoided here by also assessing the performance of cleaning agents through resin extraction and subsequent SDS‐PAGE analysis. This leads to a rigorous assessment since the absence of residuals in resin extracts consists of direct observation, but SDS‐PAGE is characterized by low throughput. However, during the screening experiments, the generated CIP results indicated rapidly the requirement of a solvent/caustic mixture. Hence, while the efficiency of these experiments could be improved, by deploying higher throughput and quantitative analytics, no bottleneck was experienced due to the small number of samples for evaluation. Conversely, when quantitative results were desired, along with higher throughput, the HCP quantitative western blotting assay was deployed. This was implemented when screening the concentrations of the NaOH and n‐propanol components in the cleaning agent and its contact time, with or without a hold, with the resin (Figure 3). The same applied when testing resin extracts from the resin re‐use experiments to determine quantitatively a significant presence of residuals as a function of cycle number. While this approach resembled the one proposed by,^[^ ^17^ ^]^ the assay used here is characterized by high sensitivity and the adopted approach allows for multiple analytics to be run, with increased levels of replication, due to extracting higher resin volumes.

The success of the screening experiments in identifying a cleaning agent was further supported by the HT scale resin re‐use experiments where even after 10 purification cycles the resin remained clean and continued to deliver a chromatography product unaffected by its re‐use. While a shorter and more efficient version of the CIP strategy could be applied (Figure 3), a more conservative approach was adopted here employing the 30% n‐propanol/1 N NaOH mixture in CIP2 with a 30 min contact time under flow. This was based on the observation that this condition was successful in cleaning columns packed with Capto Core 700 resin after they were deployed to purify the VSV∆G‐SARS‐CoV‐2 vaccine candidate and additional LVVs produced in adherent Vero cells, when none of the other tested conditions, including the isopropanol/NaOH mixture, were successful. Furthermore, such a conservative approach assisted the success of the strategy in the resin re‐use experiments and enabled the generation of results supporting the production of 10 batches of the VSV∆G‐SARS‐CoV‐2 vaccine candidate.

During the HT scale resin re‐use experiments, the cleaning of the columns was assessed by product and impurity flowthrough yields and analysis of resin extracts (Figure 5). Typically, resin re‐use experiments also employ the measurement of dynamic binding capacities, at various points between cycles, to determine the presence of persistent column fouling. This was not implemented here since RoboColumns can return variable DBCs compared to their lab‐scale counterparts.^[^ ^26^ ^]^ Moreover, the main focus of the HT investigation was to achieve a high number of re‐uses, which can be challenging with RoboColumns as they are a disposable technology; the seal at the upper frit of these columns can lose its integrity based on the type of used sample, the number of re‐uses, and also due to batch‐to‐batch variability of the columns themselves. Typically, five cycles can be completed without observing any beading of liquid at the top of these columns. Including intermittent DBC measurements in the resin re‐use experiments would therefore reduce significantly the number of cycles achieved with no beading observation. Here, re‐using the columns ten times, while applying caustics, led to beading towards the end of the cycling experiments. This and the lack of the DBC estimates at the HT scale were two driving forces for performing lab‐scale resin re‐use experiments in addition to the generation of material for UF/DF studies post‐Capto Core 700 chromatography and the desired verification of the HT scale results. Comparing the results from lab‐scale runs to those of the RoboColumns would assist to determine whether beading could lead to spurious conclusions or whether DBC measurements were required to correctly assess the performance of the devised CIP strategy. Furthermore, this comparison was critical in determining the impact of scaling RoboColumn‐based separations on a constant residence time basis and their packing quality on the generated data. Based on the observed agreement between scales, these limitations of the RoboColumn technology do not prevent them from posing as an excellent scale‐down model for designing and testing cleaning‐in‐place approaches for challenging to clean chromatography resins such as Capto Core 700.

### 3.5 Evaluation of the Capto Core 700 resin CIP strategy for the production of the VSV∆G‐SARS‐CoV‐2 vaccine candidate with multiple resin re‐use cycles

Scaling up the CIP strategy, derived from the HT scale experiments, to lab‐scale using a 20 mL pre‐packed column, led to the same conclusions as those from the HT scale resin re‐use experiments; the application of the CIP strategy delivered a chromatography product that remained unchanged across the 10 re‐use cycles (Figure 7) and the two measured intermittent BSA *DBC_10%_* values were similar to the ones obtained from columns that were not employed to purify the VSV∆G‐SARS‐CoV‐2 vaccine candidate. The resin structure was also found to be unaffected by its exposure to the cleaning agents across the 10 cycles (Figure S4, Supporting Information). The effectiveness of the CIP strategy was also demonstrated by assessing the impact of using a CCCH exposed, but not cleaned column, on BSA *DBC_10%_* measurements where it was shown that in absence of column cleaning the measured dynamic binding capacity undergoes a significant reduction (≈60%). Based on these results, the conclusion can be made that the lab‐scale data corroborate the findings from the HT scale investigations.

Focusing on the chromatography step alone assesses the impact of the CIP strategy on an intermediate process product instead of determining its impact on the final VSV∆G‐SARS‐CoV‐2 vaccine candidate product. The lack of breakthrough of HCPs (based on ELISA analysis) and hcDNA, with an increasing number of column re‐uses, (all results were below LOQ) contributed to this conclusion (Figure 7). However, the purification process includes a UF/DF step wherein the chromatography product is concentrated 50‐fold and this leads to impurity contents greater than LOQ at the final product during typical processing without any resin re‐use. This concentration step could therefore act cumulatively, across the resin re‐use cycles, and compound an elevated level of impurities in the final product. This could occur in a limiting situation wherein there is a weak breakthrough of impurities, which while it increases with the number of resin re‐use cycles is not significant enough to be observed in the chromatography product without the additional concentration offered in the UF/DF step. Hence, processing the chromatography product through the UF/DF step, which includes a concentration step adds rigor to the evaluation of the CIP strategy. This showed that re‐using the resin for 10 cycles had no impact on the final product in terms of both purity and yield (Figure 8) even in the presence of a 50‐fold concentration across the UF/DF step. This finding also indicated that more than 10 batches of the VSV∆G‐SARS‐CoV‐2 vaccine candidate could potentially be produced while CIP the Capto Core 700 column with the identified CIP strategy. While the data supporting more than 10 re‐uses would need to be generated, this prospect exemplifies the importance of identifying optimally performing cleaning conditions for chromatography columns since sub‐optimal conditions would not allow for the maximization of column re‐use cycles. The latter would be incremental in maintaining a low cost of goods with an increase in the annual production of the VSV∆G‐SARS‐CoV‐2 vaccine candidate.

The demonstrated scalability of the HT scale resin re‐use results is significant with regards to the applicability of microscale HT chromatography techniques for the development of CIP strategies. As demonstrated here, such CIP development efforts are complex and require considerable resources in terms of both time and quantities of chromatography resin and feed material. These can make their completion at a laboratory scale prohibitive, especially at the early stages of development where feed material availability can be limited. The deployment of HT chromatography techniques can mitigate these challenges since they require small amounts of materials, and their parallelization and automation enable the rapid and systematic screening of large experimental spaces to identify optimal resin cleaning conditions. While the above considers predominantly the screening of cleaning agents, demonstrating here that HT scale chromatography columns can be employed during resin re‐use experiments, and can return scalable results, support their deployment for such studies; it can lead to considerable savings in terms of used feed materials (e.g., ≈2 L at HT scale vs. ≈13 L at laboratory scale based on the approach described in Figure 1) and by offering an alternative scale for performing such long campaigns of experiments it alleviates experimental bottlenecks for laboratory‐scale development teams. These render the adoption of HT chromatography technologies an attractive alternative to laboratory‐scale feasibility studies.

## 4 CONCLUSIONS

High throughput chromatography techniques, based on RoboColumns, were employed to devise a cleaning strategy for Capto Core 700 to alleviate supply limitations met during the development of the VSV∆G‐SARS‐CoV‐2 vaccine candidate. Screens of cleaning agents revealed the need for both caustic (NaOH) and solvent (n‐propanol) at high concentrations (≥0.75 N and 30%, respectively) to yield resin extracts free of residuals. Conversely, acids, bases, and chaotropes, either deployed alone or in a combination, were not capable of cleaning the resin nor was the manufacturer recommended isopropanol and NaOH CIP solution mixture. The use of n‐propanol in the successful cleaning agent would require the CIP of a pilot and commercial‐scale columns to occur in an explosion‐proof facility. Consequently, a CIP strategy was devised employing the flushing of the column with NaOH before and after its cleaning with the solvent‐containing solution in an inactivation and storage step, respectively. This strategy was tested at a high throughput scale and was demonstrated to be effective for up to 10 resin re‐use cycles. These results were also verified at the lab scale, using a 20 mL pre‐packed column. Here, the application of the CIP strategy was found to be effective for the Capto Core 700‐based purification step and did not impact the subsequent UF/DF purification step. The final purified VSV∆G‐SARS‐CoV‐2 vaccine candidate product, delivered while cleaning and re‐using the column, was unaffected for up to 10 column re‐use cycles. The results generated in this study serve to support the application of high throughput chromatography techniques for screening, implementing, and evaluating cleaning strategies for chromatography resins. This led to demonstrating the potential elimination of a high‐risk factor, such as supply shortage, for the development of the VSV∆G‐SARS‐CoV‐2 vaccine candidate. The capability to implement these microscale studies rapidly and efficiently, along with their scalability, is therefore a valuable tool in enhancing the responsiveness of purification development teams in the face of unprecedented challenges.

## CONFLICT OF INTEREST

All authors are/were employees of Merck Sharp & Dohme LLC, a subsidiary of Merck & Co., Inc., Rahway, NJ, USA, and may potentially own stock and/or hold stock options in Merck & Co., Inc., Rahway, NJ, USA.

## Supporting information

Additional Supporting Information may be found in the online version of this article. This includes Tables S1–S7 and Figure S1–S5.

## Data Availability

The data that support the findings of this study are available from the corresponding author upon reasonable request.
