# Supplementary material for: Regeneration of Capto Core 700 resin through high throughput and laboratory scale studies and impact on production of a SARS‐CoV‐2 vaccine candidate
Source: Biotechnol J. 2022 Jul 28;17(10):2200191. doi: 10.1002/biot.202200191 (PMC9353343; doi:10.1002/biot.202200191)
Supplement: Supplementary file 1 — Additional Supporting Information may be found in the online version of this article. This includes Tables S1–S7 and Figure S1–S5. [file BIOT-17-0-s001.pdf]

SUPORTING INFORMATION

Table S1: Details of 36 cleaning in place conditions screened using Capto Core 700 RoboColumns while purifying clarified cell culture harvest to generate the SARS-CoV-2 flowthrough product pool. Each condition was implemented by applying up to three different cleaning agents in three sequential cleaning in place steps. All cleaning agents were applied for 7 column volumes, with the exception of water which was applied for one column volume.

Condition	Cleaning in place step 1 (CIP1)	Cleaning in place step 2 (CIP2)	Cleaning in place step 3 (CIP3)
1	1 N NaOH	Water	0.5 M acetic acid
2	1 N NaOH	Water	20% (v/v) ethanol; 1 M acetic acid
3	30% (v/v) isopropanol	Water	0.5 M acetic acid
4	30% (v/v) isopropanol	Water	20% (v/v) ethanol; 1 M acetic acid
5	30% (v/v) isopropanol; 1 N NaOH	Water	0.5 M acetic acid
6	30% (v/v) isopropanol; 1 N NaOH	Water	20% (v/v) ethanol; 1 M acetic acid
7	1 N NaOH	0.5 M acetic acid	None
8	1 N NaOH	20% (v/v) ethanol; 1 M acetic acid	None
9	30% (v/v) n-propanol	Water	0.5 M acetic acid
10	30% (v/v) n-propanol	Water	20% (v/v) ethanol; 1 M acetic acid
11	30% (v/v) n-propanol; 1 N NaOH	Water	0.5 M acetic acid
12	30% (v/v) n-propanol; 1 N NaOH	Water	20% (v/v) ethanol; 1 M acetic acid
13	30% (v/v) n-propanol	0.5 M acetic acid	None
14	30% (v/v) n-propanol	20% (v/v) ethanol; 1 M acetic acid	None
15	30% (v/v) n-propanol; 1 N NaOH	0.5 M acetic acid	None
16	30% (v/v) n-propanol; 1 N NaOH	20% (v/v) ethanol; 1 M acetic acid	None
17	30% (v/v) isopropanol	0.5 M acetic acid	None
18	30% (v/v) isopropanol	20% (v/v) ethanol; 1 M acetic acid	None
19	30% (v/v) isopropanol; 1 N NaOH	0.5 M acetic acid	None
20	30% (v/v) isopropanol; 1 N NaOH	20% (v/v) ethanol; 1 M acetic acid	None
21	30% (v/v) n-propanol	Water	None
22	30% (v/v) n-propanol; 1 N NaOH	Water	None
23	30% (v/v) isopropanol	Water	None
24	30% (v/v) isopropanol; 1 N NaOH	Water	None
25	0.5 M acetic acid	Water	None
26	20% (v/v) ethanol; 1 M acetic acid	Water	None
27	1 N NaOH	None	None
28	30% (v/v) n-propanol	None	None
29	30% (v/v) n-propanol; 1 N NaOH	None	None
30	30% (v/v) isopropanol	None	None
31	30% (v/v) isopropanol; 1 N NaOH	None	None
32	0.5 M acetic acid	None	None
33	20% (v/v) ethanol; 1 M acetic acid	None	None
34	8 M urea; 1 M NaCl; 0.1 citric acid; pH 2.5	None	None
35	1 M acetic acid	None	None
36	1 N NaOH	Water	None

Table S2: Spearman's rank correlation coefficient and p-values per RoboColumn (RC) between total signals at optical absorbance (280 nm), determined from the chromatography flowthrough product pool (FT), cleaning in place step 1 (CIP1), cleaning in place step 2 (CIP2) and cleaning in place step 3 (CIP3), and the number of cycles.

RC	Spearman's rank correlation coefficient				p-value			
	FT	*CIP1*	*CIP2*	*CIP3*	*FT*	*CIP1*	*CIP2*	*CIP3*
**1***	NA	NA	NA	NA	NA	NA	NA	NA
**2***	NA	NA	NA	NA	NA	NA	NA	NA
**3**	0.80	-0.40	0.80	NA**	0.3333	0.7500	0.3333	NA**
**4**	0.20	-0.40	1.00	0.77	0.9167	0.7500	0.0833	0.5000
**5**	0.11	-0.71	0.93	-0.24	0.8397	0.0881	0.0067	0.6095
**6**	0.96	-0.71	0.82	0.00	0.0028	0.0881	0.0341	1.0000
**7**	0.18	-0.81	0.93	0.70	0.6320	0.0082	0.0001	0.0240
**8**	0.66	-0.93	0.92	0.48	0.0440	0.0001	0.0005	0.1556

**, Not applicable (NA), data from a single cycle can not be used to calculate Spearman's rank correlation coefficient; **, Total signals for RC3 were all negative and hence replaced by zero making the calculation of Spearman's rank correlation*

Table S3: One way analysis of variance of anti-Spike (S) quantitative western blotting and infectivity data based on chromatography flowthrough product pool yields for RoboColumns 7 and 8 and cycles 1, 4, 7 and 10 with cycle number being the tested factor.

	Source	*SS*[a]	*DF*[b]	*MS*[c]	*F-statistic*	*p-value*
**Anti-S Yields**	*Columns*	113.11	3	37.70	1.07	0.4561
	Error	141.12	4	35.28		
	Total	254.22	7			
**Infectivity Yields**	***Source***	***SS*[a]**	***DF*[b]**	***MS*[c]**	***F-statistic***	***p-value***
	Columns	10684.79	3	3561.60	5.69	0.0632
	Error	2504.23	4	626.06		
	Total	13189.03	7			
**Infectivity Yields (cycle 1 data excluded)**	***Source***	***SS*[a]**	***DF*[b]**	***MS*[c]**	***F-statistic***	***p-value***
	Columns	1496.68	2	748.34	0.90	0.4952
	Error	2503.92	3	834.64		
	Total	4000.60	5			

a, Sum of squares; b, degrees of freedom; c, mean sum of squares

Table S4: One way analysis of variance of anti-Nucleoprotein quantitative western blotting data based on chromatography flowthrough product pool yields for each of RoboColumns (RCs) 3 - 8 with cycle number being the tested factor.

	Source	*SS*[a]	*DF*[b]	*MS*[c]	*F-statistic*	*p-value*
**RC3**	*Columns*	290.56	3	96.85	0.43	0.7382
	Error	1808.28	8	226.04		
	Total	2098.84	11			
**RC4**	***Source***	***SS*[a]**	***DF*[b]**	***MS*[c]**	***F-statistic***	***p-value***
	Columns	322.20	3	107.40	1.10	0.4042
	Error	781.93	8	97.74		
	Total	1104.13	11			
**RC5**	***Source***	***SS*[a]**	***DF*[b]**	***MS*[c]**	***F-statistic***	***p-value***
	Columns	833.82	6	138.97	1.48	0.2381
	Error	1785.05	19	93.95		
	Total	2618.86	25			
**RC6**	***Source***	***SS*[a]**	***DF*[b]**	***MS*[c]**	***F-statistic***	***p-value***
	Columns	2245.83	6	374.30	1.41	0.2593
	Error	5307.60	20	265.38		
	Total	7553.43	26			
**RC7**	***Source***	***SS*[a]**	***DF*[b]**	***MS*[c]**	***F-statistic***	***p-value***
	Columns	2594.51	9	288.28	2.01	0.0672
	Error	5009.25	35	143.12		
	Total	7603.76	44			
**RC8**	***Source***	***SS*[a]**	***DF*[b]**	***MS*[c]**	***F-statistic***	***p-value***
	Columns	3779.45	9	419.94	3.17	0.0066
	Error	4634.89	35	132.43		
	Total	8414.34	44			

a, Sum of squares; b, degrees of freedom; c, mean sum of squares

Table S5: Pairwise comparisons of RoboColumn 8 anti-Nucleoprotein quantitative western blotting data on based chromatography flowthrough product pool yields between cycles based on Tukey's method.

Comparison	Lower 95% Confidence interval	Mean difference	Upper 95% Confidence interval	p-value
Cycle 1 vs. Cycle 2	-17.95	11.71	41.37	0.9389
Cycle 1 vs. Cycle 3	-3.88	25.78	55.44	0.1343
Cycle 1 vs. Cycle 4	-33.30	-3.64	26.02	1.0000
Cycle 1 vs. Cycle 5	-28.34	0.02	28.38	1.0000
Cycle 1 vs. Cycle 6	-23.43	4.94	33.30	0.9998
Cycle 1 vs. Cycle 7	-27.97	0.40	28.76	1.0000
Cycle 1 vs. Cycle 8	-22.59	5.78	34.14	0.9994
Cycle 1 vs. Cycle 9	-16.77	11.59	39.95	0.9256
Cycle 1 vs. Cycle 10	-37.93	-9.57	18.79	0.9767
Cycle 2 vs. Cycle 3	-13.39	14.07	41.53	0.7718
Cycle 2 vs. Cycle 4	-42.81	-15.35	12.11	0.6778
Cycle 2 vs. Cycle 5	-37.74	-11.69	14.37	0.8776
Cycle 2 vs. Cycle 6	-32.82	-6.77	19.28	0.9963
Cycle 2 vs. Cycle 7	-37.36	-11.31	14.74	0.8965
Cycle 2 vs. Cycle 8	-31.98	-5.93	20.12	0.9986
Cycle 2 vs. Cycle 9	-26.17	-0.12	25.93	1.0000
Cycle 2 vs. Cycle 10	-47.33	-21.28	4.77	0.1913
*Cycle 3 vs. Cycle 4	-56.88	-29.42	-1.96	0.0277
Cycle 3 vs. Cycle 5	-51.81	-25.76	0.29	0.0547
Cycle 3 vs. Cycle 6	-46.90	-20.84	5.21	0.2128
Cycle 3 vs. Cycle 7	-51.44	-25.38	0.67	0.0613
Cycle 3 vs. Cycle 8	-46.06	-20.00	6.05	0.2592
Cycle 3 vs. Cycle 9	-40.24	-14.19	11.86	0.7075
*Cycle 3 vs. Cycle 10	-61.40	-35.35	-9.30	0.0020
Cycle 4 vs. Cycle 5	-22.39	3.66	29.71	1.0000
Cycle 4 vs. Cycle 6	-17.48	8.58	34.63	0.9802
Cycle 4 vs. Cycle 7	-22.02	4.04	30.09	0.9999
Cycle 4 vs. Cycle 8	-16.64	9.42	35.47	0.9640
Cycle 4 vs. Cycle 9	-10.82	15.23	41.28	0.6223
Cycle 4 vs. Cycle 10	-31.98	-5.93	20.12	0.9986
Cycle 5 vs. Cycle 6	-19.65	4.91	29.48	0.9995
Cycle 5 vs. Cycle 7	-24.19	0.37	24.94	1.0000
Cycle 5 vs. Cycle 8	-18.81	5.75	30.32	0.9983
Cycle 5 vs. Cycle 9	-13.00	11.57	36.13	0.8443
Cycle 5 vs. Cycle 10	-34.15	-9.59	14.97	0.9426
Cycle 6 vs. Cycle 7	-29.10	-4.54	20.02	0.9997
Cycle 6 vs. Cycle 8	-23.72	0.84	25.40	1.0000
Cycle 6 vs. Cycle 9	-17.91	6.65	31.22	0.9949
Cycle 6 vs. Cycle 10	-39.07	-14.51	10.06	0.6091
Cycle 7 vs. Cycle 8	-19.18	5.38	29.94	0.9990
Cycle 7 vs. Cycle 9	-13.37	11.19	35.76	0.8674
Cycle 7 vs. Cycle 10	-34.53	-9.97	14.60	0.9285
Cycle 8 vs. Cycle 9	-18.75	5.81	30.38	0.9982
Cycle 8 vs. Cycle 10	-39.91	-15.35	9.22	0.5340
Cycle 9 vs. Cycle 10	-45.72	-21.16	3.40	0.1417

**, Significant difference in yields*

Table S6: One way analysis of variance of ELISA HCP data from the final product post ultrafiltration/diafiltration between cycles 1, 4, 7 and 10 with cycle number being the tested factor.

Source	*SS*[a]	*DF*[b]	*MS*[c]	*F-statistic*	*p-value*
Columns	0.03	3	0.01	59.21	0.0009
Error	0.00	4	0.00		
Total	0.03	7			

a, Sum of squares; b, degrees of freedom; c, mean sum of squares

Table S7: Pairwise comparisons of ELISA HCP data from the final product post ultrafiltration/diafiltration between cycles 1, 4, 7 and 10 based on Tukey's method.

Comparison	Lower 95% Confidence interval	Mean difference	Upper 95% Confidence interval	p-value
Cycle 1 vs. Cycle 4	-0.09	-0.04	0.01	0.0777
Cycle 1 vs. Cycle 7	-0.19	-0.14	-0.09	0.0012
Cycle 1 vs. Cycle 10	-0.04	0.01	0.06	0.9443
Cycle 4 vs. Cycle 7	-0.14	-0.09	-0.04	0.0052
Cycle 4 vs. Cycle 10	0.00	0.05	0.10	0.0499
Cycle 7 vs. Cycle 10	0.09	0.14	0.19	0.0010

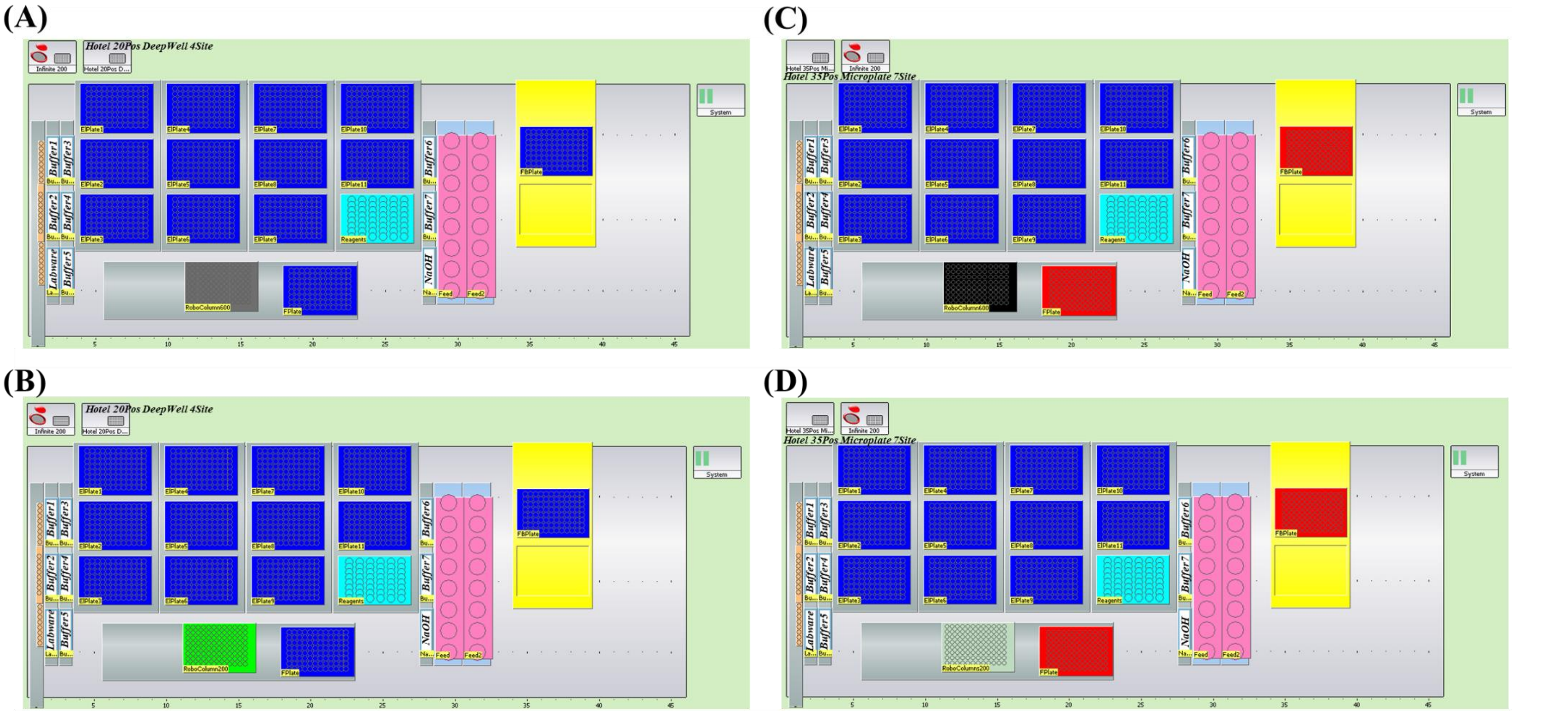

Figure S1: Deck layout of the employed Tecan EVO 150 robotic station used to run RoboColumn based experiments: (A) 600 µL RoboColumns (*RoboColumn600*) with 2 mL deep well 96 well plates for fraction collection (*FPlate*); (B) 200 µL RoboColumns (*RoboColumn200*) with 2 mL deep well 96 well plates for fraction collection (*FPlate*); (C) 600 µL RoboColumns (*RoboColumn600*) with 360 µL 96 well microplates for fraction collection (*FPlate*); (D) 200 µL RoboColumns (*RoboColumn200*) with 360 µL 96 well microplates for fraction collection (*FPlate*). The robot was equipped with a 8-channel liquid handling arm (left-hand side) and a robot manipulator arm (RoMa) (right-hand side). In (A) – (C): (i) *ElPlate1 – ElPlate11* are 2 mL deep well 96 well plates containing elution buffers; (ii) *Reagents* is a 8 mL 48 well deep well plate containing equilibration, feed, wash, strip (or post-CIP flush here), CIP, and storage solutions in columns 1 – 6, respectively; *Feed* and *Feed2* are 50 mL Falcon tubes containing up to 8 different feeds, each at ~ 100 mL, and were used for high loadings instead of placing the feeds in the *Reagents* plate; *FBPlate* denotes the location of 96 well plates (deep well or microplates) containing buffers (i.e., blank plates) which were prepared by aliquoting into them solutions from labwares *ElPlate1 – ElPlate11* and *Reagents*; 100 mL troughs *Buffer1 – Buffer7*, *Labware* were empty and *NaOH* was filled with concentrated NaOH solution which was used to clean the tips. 100 mL troughs are placed on *Trough 3Pos 25+100ml* carriers. *ElPlate1 – ElPlate11* and *Reagents* are placed on *MP 3Pos Flat* carriers with custom definitions to allow the placement of the Te-Shuttle module in front of them. *Feed* and *Feed2* are placed on *Falcon 8Pos 50mL* carriers. *FBPlate* is placed on the loading bay of the integrated Agilent Velocity 11 VSpin with access centrifuge. RoboColumns are placed on the Te-Chrom module and its height was adjusted to allow the collection of fractions in microplates or deep well plates. *FPlate* is placed on the Te-Shuttle (Transfer position) when a new fraction plate is to be generated. *FPlate* and *FBPlate* were handled by the RoMa which also moved the plates from the hotels to the robot's deck. The hotels are comprised of multiple *Hotel 9Pos* carriers. These were configured either as a single hotel with 7 z-sites and 5 x-sites (*Hotel 35Pos Microplate 7Site* carrier in A, B) for microplates, or as a single hotel with 4 z-sites and 5 x-sites (*Hotel 20Pos DeepWell 4Site* carrier in C, D) for deep well plates. The *Infinite 200* carrier is the Tecan Infinite M1000 Pro reader placed at the right-hand side of the robot's deck (grid 45).

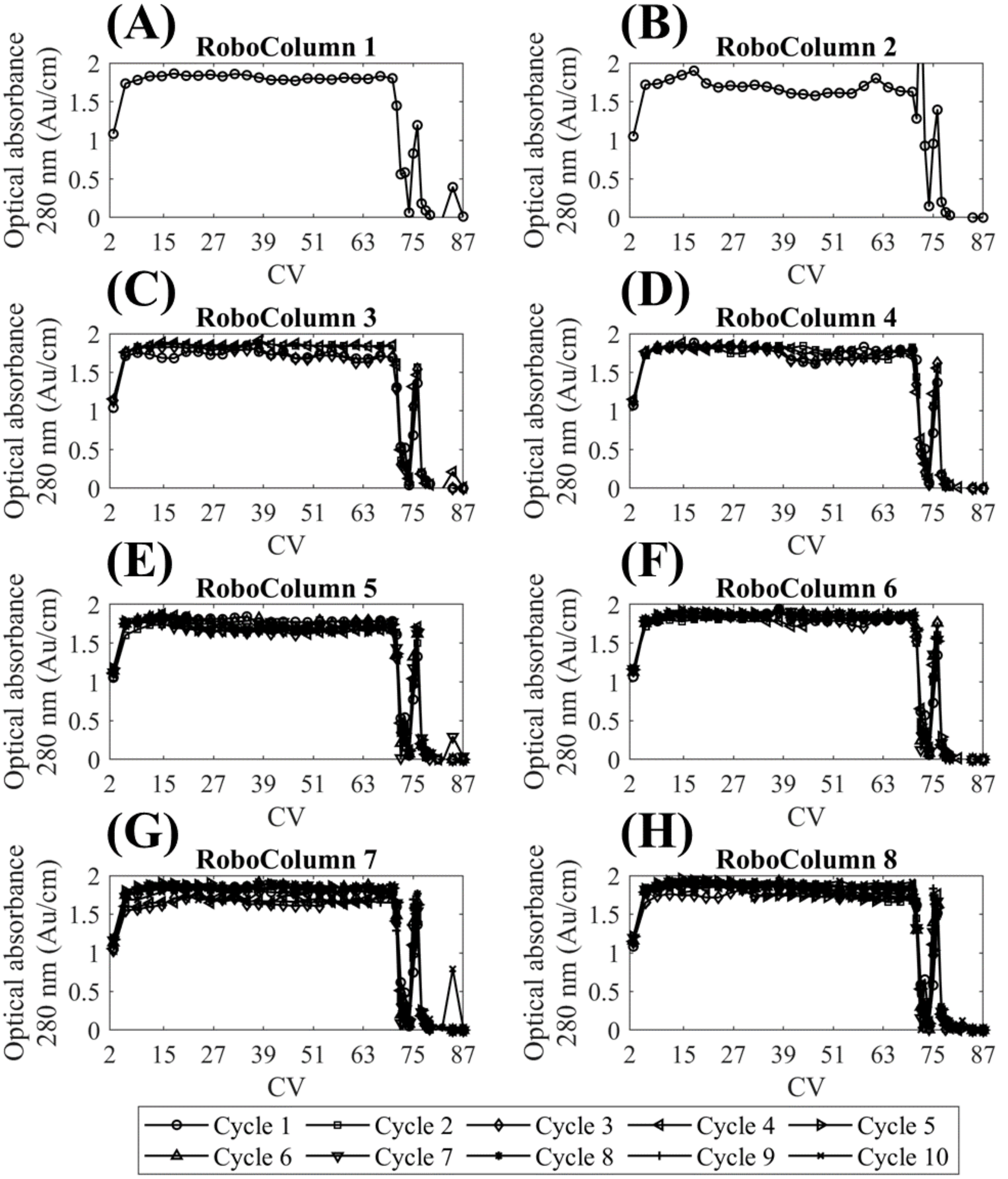

Figure S2: Optical absorbance at 280 nm plotted against column volumes (CVs) across the ten re-use cycles for RoboColumns 1 – 8 in (A) – (H), respectively. In (A) – (H), lines with markers (○), (□), (◊), (◁), (▷), (△), (▽), (✳), (+), (×) correspond to resin re-use cycles 1 – 10, respectively.

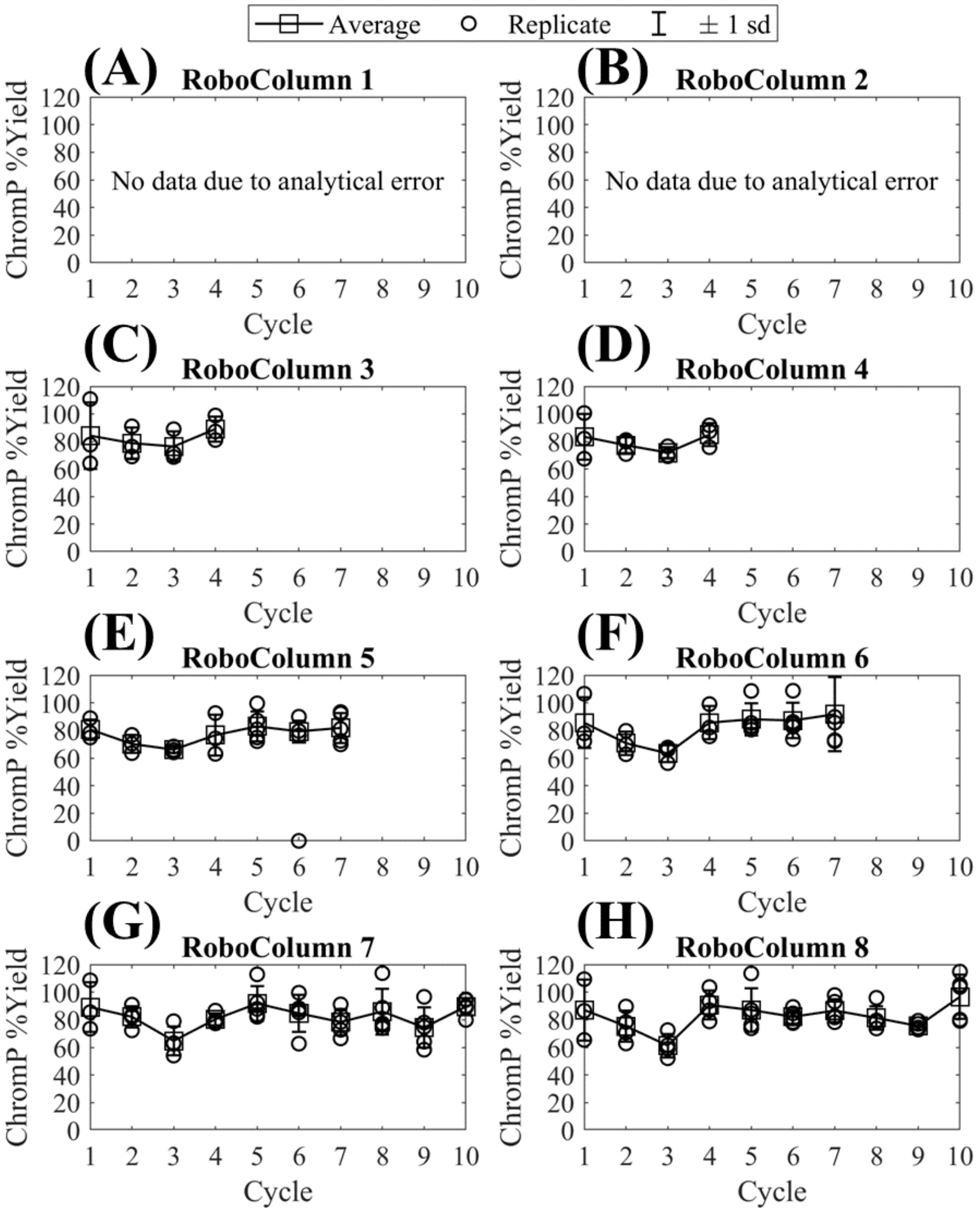

Figure S3: Average high throughput chromatography flowthrough product pool yields as a function of resin re-use cycle based on anti-Nucleoprotein (N) protein quantitative western blotting (□) data for RoboColumns 1 – 8 in (A) – (H), respectively. In (A) – (H), open circle (○) symbol represents analytical replicates and error bars correspond to ± 1 standard deviation (sd).

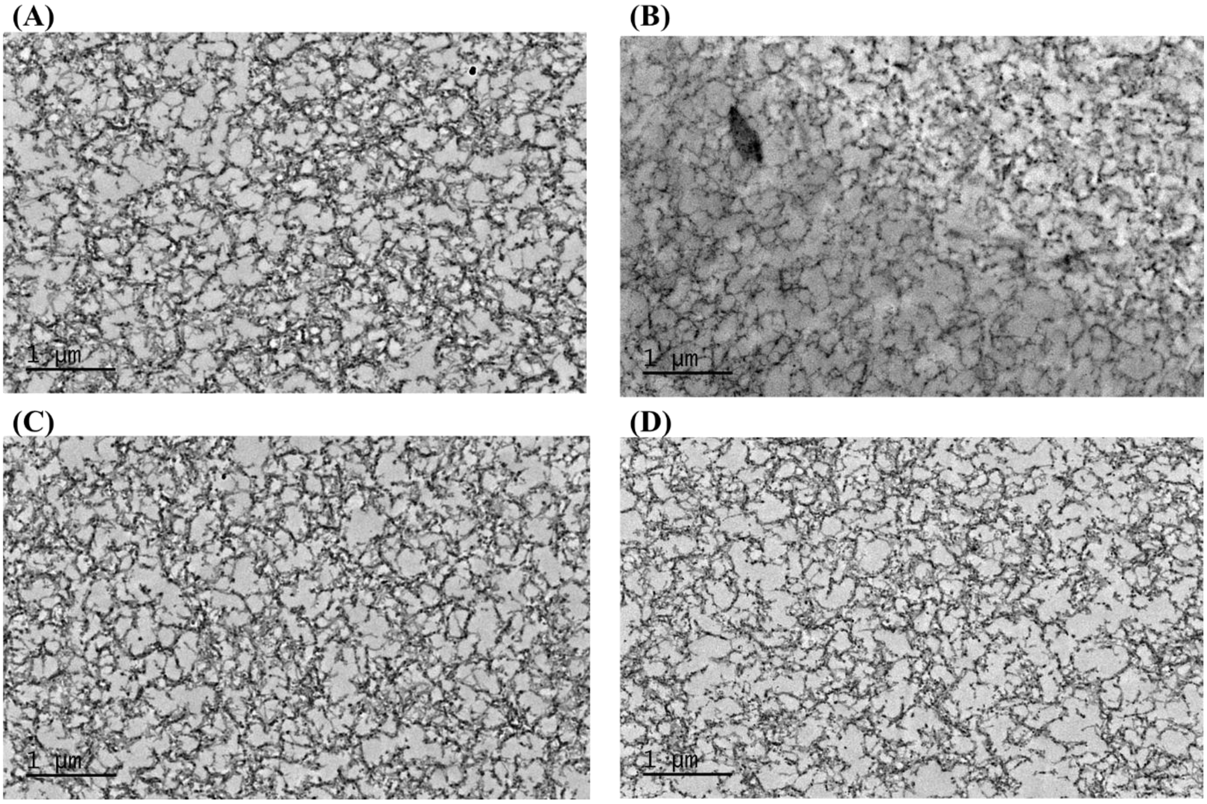

Figure S4: Transmission electron microscopy images of: (A) Fresh and unused Capto Core 700; (B) – (D) resin samples collected at the end of the ten resin re-use cycles at lab scale from the top third, middle third, and the bottom third of the 20 mL Capto Core 700 column, respectively. In (A) – (D) scale bar is 1 µm and the magnification was set at 23000X. Shown images were taken at the interior of the resin's beads.

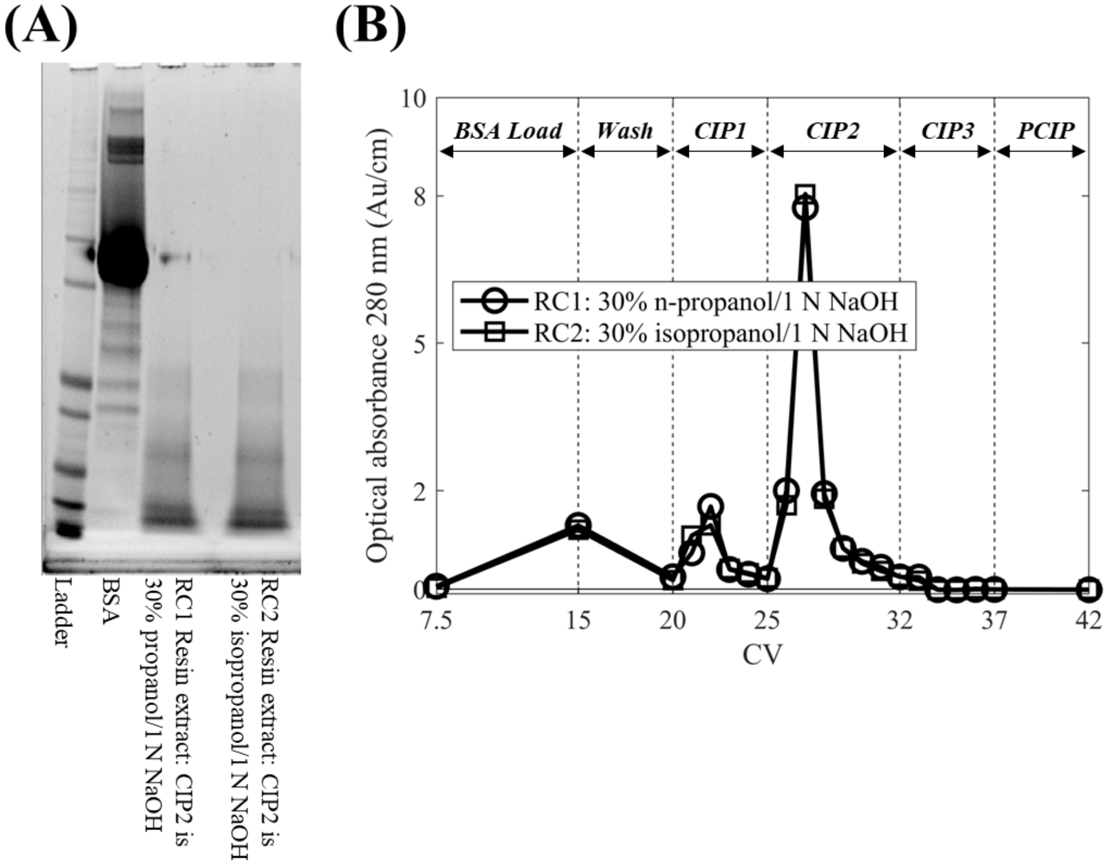

Figure S5: Evaluation of three step (i.e., CIP1, CIP2, CIP3) cleaning in place (CIP) strategy for cleaning Capto Core 700 resin exposed to a bovine serum albumin (BSA) (2.74 g/L) load with two different solutions in CIP2, each tested in a separate 600 µL RoboColumn (i.e., RC1 and RC2): (A) SDS-PAGE analysis of the loaded BSA and the RC1 and RC2 resin extracts, each obtained by using a 30% n-propanol/1 N NaOH or a 30% isopropanol/1 N NaOH solution in CIP2; (B) Optical absorbance at 280 nm as a function of column volumes (CVs). In (B), the (○) and (□) symbols denote the use of 30% n-propanol/1 N NaOH and 30% isopropanol/1 N NaOH solutions in CIP2 for RC1 and RC2, respectively. Double-headed arrows denote the beginning and end of each phase for RC1 and RC2. PCIP refers to the post-CIP flush of the columns. The buffers and solutions used in the loading, washing, cleaning in place, and flushing the column post its CIP match those used in the lab scale BSA dynamic binding capacity measurement experiments. The same applied to the residence times per phase.
